# Towards a morphological metric of assemblage dynamics in the fossil record: a test case using planktonic foraminifera

**DOI:** 10.1098/rstb.2015.0227

**Published:** 2016-04-05

**Authors:** Allison Y. Hsiang, Leanne E. Elder, Pincelli M. Hull

**Affiliations:** Department of Geology and Geophysics, Yale University, P.O. Box 208109, New Haven, CT 06520-8109, USA

**Keywords:** automated three-dimensional morphometrics, planktonic foraminifera, community ecology, community morphopology, macroecology, virtual palaeontology

## Abstract

With a glance, even the novice naturalist can tell you something about the ecology of a given ecosystem. This is because the morphology of individuals reflects their evolutionary history and ecology, and imparts a distinct ‘look’ to communities—making it possible to immediately discern between deserts and forests, or coral reefs and abyssal plains. Once quantified, morphology can provide a common metric for characterizing communities across space and time and, if measured rapidly, serve as a powerful tool for quantifying biotic dynamics. Here, we present and test a new high-throughput approach for analysing community shape in the fossil record using semi-three-dimensional (3D) morphometrics from vertically stacked images (light microscopic or photogrammetric). We assess the potential informativeness of community morphology in a first analysis of the relationship between 3D morphology, ecology and phylogeny in 16 extant species of planktonic foraminifera—an abundant group in the marine fossil record—and in a preliminary comparison of four assemblages from the North Atlantic. In the species examined, phylogenetic relatedness was most closely correlated with ecology, with all three ecological traits examined (depth habitat, symbiont ecology and biogeography) showing significant phylogenetic signal. By contrast, morphological trees (based on 3D shape similarity) were relatively distantly related to both ecology and phylogeny. Although improvements are needed to realize the full utility of community morphometrics, our approach already provides robust volumetric measurements of assemblage size, a key ecological characteristic.

## 1. Introduction

Speciation and extinction are population-level processes with global effects on biodiversity. A species ceases to be when the last individual of the last population dies, and a new species arises when two previously connected populations become sufficiently isolated [1]. This being the case, assemblage dynamics should provide the most direct test of various regulators of biodiversity—be they environmental, biological or neutral—but this is almost never done in deep time. The vast majority of taxa simply do not have fossil records up to the task. Instead, questions of biodiversity dynamics and their drivers are typically addressed at the species level (or higher) in one of two ways [2]: (i) fitting models of diversification to modern and, rarely, fossil phylogenies (e.g. [3–7]) and (ii) assessing correlates of global diversity dynamics from fossil compilations and databases, like the Paleobiology Database (PBDB) (e.g. [8–11]). In all cases, the role of various regulators is inferred from their end-effect on phylogenetic structure or standing diversity, with varying degrees of theoretical robustness to the inference (as discussed in [6,12]). For those few taxa that do have excellent fossil records, like marine microfossils [13], assemblage-level studies of populations through time offer the exciting possibility of testing evolutionary mechanisms hypothesized from other data types (see [14,15] for a macrofossil example).

Unfortunately, even for those rare taxa with the spatial and temporal coverage needed to track population dynamics through geological time, there still remains a daunting data-collection problem. Measuring proxies of environmental and biological effects on numerous populations is extremely time intensive. As a result, most population-level work to-date has focused on environmental regulators of species abundance or range (e.g. [16]), or evolutionary dynamics within a lineage [17–19]. The tendency to investigate environmental regulators of population dynamics alone arises, in part, because environmental proxies are relatively quick and easy to measure, whereas high-throughput phenotypic methods have lagged for biological drivers. This study is aimed directly at this biological data-collection problem using a palaeontological model taxon: planktonic foraminifera.

Planktonic foraminifera are marine protists with calcium carbonate shells (technically, ‘tests’). Found throughout the global ocean today and abundantly for (roughly) the past 150 Myr, their tests rain to the sea floor on death and, in many regions, are preserved in near-continuously accumulating deposits [20,21]. Their abundance [20], the potential to measure multiple environmental proxies directly from their test geochemistry [21], and the recent completion of a phylogeny for Cenozoic macroperforate species (the major clade of planktonic foraminifera) [22] all contribute to the growing utility of planktonic foraminifera for understanding macroevolution [18,19,23–25]. Because planktonic foraminifera are a central tool in the field of palaeoceanography (the study of ancient oceans) [20,21,26], detailed records of environmental conditions already exist in many locations and time intervals over the past 66 Myr (e.g. [27–29]). Comparable biotic data are often lacking, however, and this disconnect remains a conspicuous hindrance to our ability to understand the feedbacks between biotic and abiotic processes in macroecology and macroevolution.

Here, we develop the methods for, and provide an initial view of the utility of, high-throughput semi-three-dimensional (semi-3D) geometric morphometrics as a means for rapidly tracking biotic dynamics through time. This approach rests on the assumption that the morphology of individuals, including their body size, reflects some combination of shared evolutionary history, functional ecology and individual variation. Several lines of evidence suggest that this may be the case in planktonic foraminifera. Importantly, planktonic foraminifera are well known for exhibiting iterative evolution of gross morphology. In multiple cases, complex morphologies including flat discoidal or peaked pyramidal morphologies, finger-like chambers and sharp edges (known as keels) have independently evolved from simple, globular ancestors [30–32]. Although functional morphology is poorly understood (see discussions in [33,34]), this ubiquity of convergent evolution in planktonic foraminifera and the correlation, in some cases, of morphology and life history support the inference of a functional role for gross morphology. If this is the case, then the morphological similarity of communities might provide a measure of functional similarity—an approach sometimes called ‘ecometrics’ and related to the burgeoning field of functional trait ecology [35].

That said, the relationship between diversity, functional diversity and morphological diversity is not straightforward (e.g. [36,37]), and morphological measures of community dynamics would necessarily remain just that—morphological measures—without thoughtful exploration and calibration. Even so, for deep time studies, community morphology provides a particularly promising means of assessing biotic dynamics because morphology provides a common ruler to compare across assemblages with entirely different species compositions [35,38]. For planktonic foraminifera, and many other groups, direct measures of morphology solve a second problem related to the common occurrence of morphologically intermediate individuals that lie between named taxa (for examples of morphological variation, see: [18,19,39]). Morphologically intermediate individuals can provide direct evidence of evolution in action, but their importance and implications are missed when morphologically intermediate taxa are shoehorned into named species-categories.

In planktonic foraminifera, it is clear that shared evolutionary history and factors unique to individuals influence morphology. Planktonic foraminiferal genera are often readily recognizable by shared, derived gross morphological characteristics, providing support for the inference that morphology must partially reflect the evolutionary relatedness of taxa. In some cases, speciation or evolutionary transitions have occurred across habitat types with relatively minor morphological change. Such instances include depth parapatry within a pseudo-cryptic species [40], abrupt ecological change within a gradual morphological series [41] and the occurrence of deep-water morphologies in shallow water habitats [42,43], and *vice versa* (as discussed in [33]). Finally, at an individual level, the availability of various resources (like light, food, temperature and oxygen) can profoundly influence adult size, shape and wall structure (e.g. thickness and porosity) [44–47].

The intent of this study is to lay the groundwork for the application of community morphology as a measure of population dynamics in planktonic foraminifera, although the underlying methods are fully applicable, and currently being used in-house, in macrofossils as well. To this end, we have:

— developed a pipeline to extract 3D data from light images (semi-3D morphometrics),
— compared morphological space represented by meshes from 16 species extracted using slow, but highly resolved, computed tomography (CT) (full-3D) and our new method (semi-3D),
— examined the relationship between morphology (full- and semi-3D), ecology and phylogeny in those 16 species,
— demonstrated the utility of our method for rapidly collecting traits like volume and surface area,
— investigated the trade-offs in data density and quality in 3D morphometrics and
— explored the potential of assemblage-wide ecometrics with four modern planktonic foraminiferal assemblages in the North Atlantic.

This work provides a new set of image-processing programs for extracting semi-3D data from light images, while highlighting the potential (and problems) of our approach, and provides a first exploration of the relationship between gross morphology (as measured with geometric morphometrics), ecology and phylogeny in 16 species of modern planktonic foraminifera.

## 2. Material and methods

### (a) Specimen sources

Two fundamentally different types of 3D data are used in this study: full-3D meshes from X-ray CT (CT scans) and semi-3D meshes from reflected light microscopy. The two data types have complementary strengths and weaknesses. CT captures the full 3D shape of planktonic foraminifera, including internal structure (although it should be noted that our subsequent analyses use only exterior shape from the CT scans), but is relatively slow to collect. By contrast, our light microscopic method (as detailed below) is very fast, but only captures exterior 3D shape from a single viewpoint (hence, semi-3D). In this study, we use the CT scans as a reference point to test the relationship between 3D morphology, ecology and phylogeny, and to test the relative information captured with the rapid semi-3D methods that we develop and introduce here.

Thirty-nine full-3D meshes from X-ray CT were obtained from Tohoku University Museum's e-Foram Stock database [48], representing 19 extant species of planktonic foraminifera (electronic supplementary material, table S1). The Tohoku University specimens were imaged with high-resolution X-ray CT scans (generated using ScanXmate-E090; Comscantecno Corporation) at 5 µm resolution [48]. We refer, hereafter, to these complete specimens as the Tohoku University specimens or as full-3D data.

**Figure 1.**
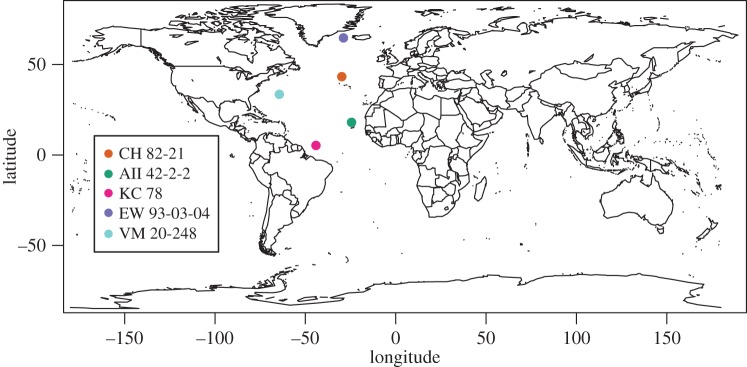
Map of the five Atlantic coretops sites used in this study.

We generated new, semi-3D meshes of 281 individuals identified to species level, representing 24 species of extant planktonic foraminifera. The complete list of all specimens used in this study (including database, catalogue number and species) is presented in electronic supplementary material, table S1. Five coretop locations in the North Atlantic were used as focal sites for this study to maximize taxonomic diversity/disparity at a community level and to explore the size distributions obtained from two-dimensional (2D) versus 3D imaging (figure 1). An additional 116 planktonic foraminifera from these sites, not identified to the species level, were included for community comparison purposes. The sites are: KC 78 (5°16′01″ N and 44°07′59″ W), CH 82-21 (43°29′17″ N and 29°49′48″ W), EW 93-03-04 (64°43′ N and 28°55′ W), VM 20-248 (33°30′ N and 64°24′ W) and AII 42-2-2 (18°01′59″ N and 24°27′ W). An example slide image for each site is available via the Yale University Peabody Museum of Natural History (YPM) online database (KE EMu) via the listed catalogue numbers and the Division of Invertebrate Paleontology Portal (http://peabody.yale.edu/collections/search-collections?ip). Full raw images are available upon request, as no public repository exists, free of charge, for large image files. YPM catalogue numbers for the five focal sites are: KC 78 (IP.307630–IP.307634), CH 82-21 (IP.307625–IP.307628), EW 93-03-04 (IP.307636–IP.307640), VM 20-248 (IP.307715) and AII 42-2-2 (IP.307647–IP.307651). To improve species coverage, additional exemplar individuals were included from 12 species: *Truncorotalia crassaformis* (IP.307720), *Pulleniatina obliquiloculata* (IP.307727), *Truncorotalia truncatulinoides* (IP.307733), *Neogloboquadrina dutertrei* (IP.307862), *Globigerinella siphonifera* (IP.307863), *Globorotalia tumida* (IP.307749), *Menardella menardii* (IP.307754), *Menardii fimbriata* (IP.307754), *Sphaeroidinella dehiscens* (IP.307757), *Hirsutella hirsuta* (IP.307760), *Globigerinoides conglobatus* (IP.307763, IP.307764), *Globoconella inflata* (IP.307767), *Candeina nitida* (IP.307772, IP.307773) and *Globigerinella calida* (IP.307865). Note that although only 281 specimens were identified to species level for morphological analyses, a total of 9681 individual planktonic foraminifera were used to compare body size distributions across four focal sites (no. of individuals by site: 1768 from KC 78; 2879 from CH 82-21; 3034 from EW 93-03-04; and 2000 from AII 42-2-2).

Specimen completeness and species identification were conducted by eye by PMH and LEE. Species were identified following the naming scheme of Aze *et al.* [22], and taxonomic concepts of Kennett & Srinivasan where applicable [49], to facilitate direct comparisons with the macroperforate phylogeny. Exceptions to the Aze *et al.* [22] species-naming scheme were as follows:

(i)*Truncorotalia*: All *Truncorotalia* were identified as either *T. truncatulinoides* or *T. crassaformis*. Aze *et al*. [22] recognizes six extant *Truncorotalia*. The first two (*T. crassaformis* and *T. oceanica*) are allied with the *T. crassaformis* complex and the remaining four (*T. cavernula, T.excelsa, T. pachytheca* and *T. truncatulinoides*) with the *T. truncatulinoides* complex, with morphological and genetic definitions varying among authors (e.g. [22,50–53]).
(ii)*Pulleniatina*: All *Pulleniatina* were identified as *P. obliquiloculata*, ignoring the possibility of *P. finalis*. This was a practical decision: from the common imaging angle (umbilical), these taxa are not readily distinguished.
(iii)*Globigerinoides triloba*: All *G. triloba* (morphospecies concept) were classified as *Trilobatus sacculifer* following genetic evidence for a single modern species in this morphologically variable taxa [54,55].
(iv)*Neogloboquadrina*: We recognized *N. incompta*, in addition to the *N. pachyderma* and *N. dutertrei* of Aze *et al*. [22], given widespread support for the genetic separation of this readily identified taxa [56,57].

In short, we generally favoured a ‘clumped’ taxonomic approach in naming (excepting *N. incompta*), allowing morphological variation to highlight differences among closely related lineages. Planktonic foraminifera morphospecies commonly harbour a few (pseudo-)cryptic genetic species [58,59], but the degree of splitting still varies among authors and is still being resolved [57].

We analyse the relationship between morphology, phylogeny and ecology, in the 15 species of macroperforate foraminifera in common between the full- and semi-3D datasets. These 15 species are listed in table 1, along with their ecological characteristics. The 15 macroperforate species and one microperforate species, *C. nitida*, were also used to assess the relative morphological information contained in full- and semi-3D meshes.

**Table 1.** Ecological traits of overlapping macroperforate planktonic foraminifer species.

scientific name	symbiont type	habitat depth	geographical range	refs
*Globigerina bulloides*	none	mixed layer	mid-latitudes	[60–66]
*Globigerinella siphonifera*	chrysophytes	mixed layer/thermocline	low–mid latitudes	[40,60,63,67–71]
*Globigerinoides conglobatus*	dinoflagellates	mixed layer	low latitudes	[60,64,68,69]
*Globigerinoides ruber*	dinoflagellates	mixed layer	low latitudes	[21,60,64,68,69,71–73]
*Globoconella inflata*	chrysophytes	thermocline	low–high latitudes	[21,22,60,68,69,74]
*Globorotalia tumida*	none	thermocline/sub-thermocline	low latitudes	[21,22,60,63,68,69,73,75,76]
*Hirsutella hirsuta*	none	thermocline/sub-thermocline	low–mid latitudes	[21,60,68,69,74]
*Menardella menardii*	chrysophytes	thermocline	low latitudes	[21,60,63,68,71,74]
*Neogloboquadrina dutertrei*	chrysophytes	mixed layer/thermocline	low latitudes	[22,60,64,68,69,71,74,77,78]
*Neogloboquadrina pachyderma*	none	mixed layer/thermocline	low–high latitudes	[21,60,68–70,74,79]
*Pulleniatina obliquiloculata*	chrysophytes	mixed layer/thermocline	low latitudes	[21,60,68,69,71,74,79]
*Sphaeroidinella dehiscens*	dinoflagellates	thermocline	low latitudes	[22,60,68,80]
*Trilobatus sacculifer*	dinoflagellates	mixed layer	low latitudes	[21,60,64,68,69,71–73]
*Truncorotalia crassaformis*	none	sub-thermocline	low–mid latitudes	[22,68,69,71,79]
*Truncorotalia truncatulinoides*	none	sub-thermocline	low latitudes	[21,22,60,62,68,69,71,74,79]

### (b) Slide preparation and imaging for semi-3D data

Semi-3D data were collected from five sites (figure 1) and our species exemplar slide collection. For each of the five sites, a micropalaeontological split of the greater than 150 µm fraction was taken with a target sample size of 5000 individuals per site. Splits were scattered, oriented to an umbilical view, and glued to plain black micropalaeontogical slides. Approximately four slides were used per site to accommodate the roughly 5000 individuals in the split. Slides were imaged on a Leica Microsystems DM6000M compound microscope with transmitted light, a 5× objective and a 5-megapixal Leica DFC450 digital camera, using a 64-bit beta version of the controlling Surveyor Software. Each slide was scanned in a series of tiles in the *x*- and *y*-dimensions to cover the full length and width of the slide (using the automated stage). For each *x*–*y* tile, the automatic drive focus took a series of images at different heights (with a prescribed *z*-step) to capture the full-depth dimension of the fossils.

Each slide scan was saved as 32 bigTiff files, with the first 31 files capturing a single depth slice of the scan (*x*- and *y*-tiles composited). The 32nd bigTiff file is a 2D extended depth of focus image of the slide. A *z*-step size of 31.1 µm was used to account for the depth of focus of the 5× objective. This step size sets the limit of our ability to resolve shape in the *z*-dimension. Pixel size in the *x*- and *y*-dimensions was 0.975 µm. Microscope settings were the same for the exemplar slide scanning, with the only difference being the number of individuals scanned per slide (several individuals rather than thousands of individuals).

The reproducibility of the 3D-mesh extraction pipeline to variation in the orientation of mounted specimens and imagining problems (e.g. image tiling, glare) was tested using a single representative of each of the following eight species: *Trilobatus sacculifer* (IP.307747), *G. tumida* (IP.307749), *P. obliquiloculata* (IP.307751), *N. dutertrei* (IP.307753), *Orbulina universa* (IP.307756), *Globigerinoides ruber* (IP.307758), *T. truncatulinoides* (IP.307762) and *G. siphonifera* (IP.307765). Each individual was (re-)mounted five times and (re-)imaged in five different settings (per mount) with varying field of views and white balance/shade correction settings. The five imaging tests conducted per mount included: (i) a vertical image seam along specimen (e.g. specimen at the joint of a left and right image tile); (ii) a horizontal image seam along specimen (e.g. specimen at the joint of a upper and lower image tile); (iii) a centred specimen (single image tile) with the same white balance and shade correction for tests #1–3; (iv) a centred specimen (single image tile) with a readjusted white balance/shade correction; and (v) a centred specimen (single image tile) with a readjusted white balance/shade correction. In total, 25 images (five mounts per specimen and five imaging tests per mount) of the same individual were included for each species listed above, resulting in a final set of 200 reproducibility test ‘individuals’.

### (c) Preliminary image processing for semi-3D data

The *segment* (v. 1.10) and *focus* (v. 1.10) functions from the image-processing module of the *AutoMorph* software package (current software available on GitHub at https://github.com/HullLab) were used to extract individual objects from the scanned slides. *segment* chops slide scans up into individual objects and *focus* generates an extended depth of focus image for each object.

For *segment,* a black/white thresholding value of 0.18 was used for all slides except for CH 82-21 (threshold = 0.14), KC 78 (threshold = 0.20) and EW 93-03-04 (threshold = 0.20). The absolute thresholding value is unimportant for the analyses that follow, and are varied by slide to optimally segment out all foraminifera (common errors include segmentation of background glare, edge clipping of transparent or darkened individuals, etc.). The size range filter for a valid object was set to 125–2000 µm (width).

*focus* was run using the *Zerene Stacker* software [81] to generate a best extended depth of focus image (EDF) per object. *Zerene Stacker* settings included brightness correction between frames, automatic order of images (e.g. focus stacking begins on image with the narrowest field of view), default values of the estimation radius (10) and smoothing radius (5), a contrast threshold of 25%, and a grit suppression algorithm to reduce noise and pixellation during image stacking.

*run2dmorph* (v. 1.07, also available on GitHub), another module of the *AutoMorph* package, was run on the focused images to extract 2D morphology (e.g. outlines and corresponding coordinates) and shape parameters (e.g. major and minor axes length, enclosed area, eccentricity, rugosity, perimeter and aspect ratio) for body size analyses. All three processes (*segment*, *focus*, *run2dmorph*) were performed on the Department of Geology and Geophysics Tide server at Yale University.

### (d) Extraction of semi-3D mesh

A new 3D mesh extraction module, *run3dmorph*, was written by AYH as part of the *AutoMorph* software package to extract semi-3D meshes and object volumes from the objects (in this case, planktonic foraminifera) after preliminary image processing via *segment* and *focus*. A beta version of *run3dmorph* is available on GitHub at this time, with early adopters encouraged to check for updates (https://github.com/HullLab).

**Figure 2.**
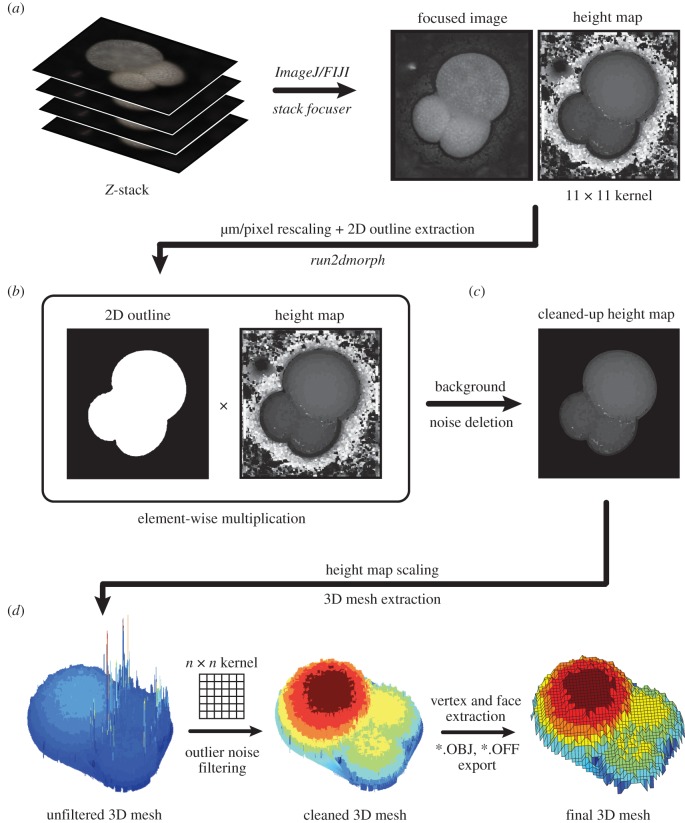
Visual pipeline illustrating the steps involved in 3D mesh extraction using the *run3dmorph* software. (*a*) *Z*-stacks of each individual object (taken at varying focal planes of known height above the object) are processed using the *Stack Focuser* plug-in for *ImageJ/FIJI*, resulting in a focused image of the object and a height map (built using an 11 × 11 pixel kernel size). (*b*) The focused image and height map are rescaled such that each pixel has a height and width of 1 µm, and the 2D outline of the focused image is extracted using the *run2dmorph* software (see https://github.com/Hull-Lab). Each pixel of the binary 2D outline image is then multiplied against the corresponding pixel in the height map (element-wise multiplication). This effectively deletes background noise and results in a cleaned-up height map (*c*). The greyscale value of each pixel in the height map is then used, in conjunction with the distance between each *z*-stack slice, to back-calculate the real-world height of each pixel and generate an unfiltered 3D mesh (*d*). High and low outlier noise is then filtered from the 3D mesh using a custom neighbourhood pixel-averaging algorithm (using a user-defined *n* × *n* pixel kernel, where *n* is a positive odd integer). Vertex and face coordinates are then extracted from the cleaned 3D mesh and outputted in standard 3D ASCII formats (OBJ and OFF).

The generalized pipeline for the 3D mesh extraction is shown in figure 2. First, a greyscale EDF image and height map is generated for each individual using the *Stack Focuser* plugin [82] for *ImageJ* [83,84] and *FIJI* [85]; an 11 × 11 pixel kernel was used for the height map generation (figure 2*b*). The 3D mesh is then extracted from a cleaned height map (figure 2*c,d*) using a series of custom *MATLAB* scripts, which require *MATLAB* version 2015b or above [86].

**Figure 3.**
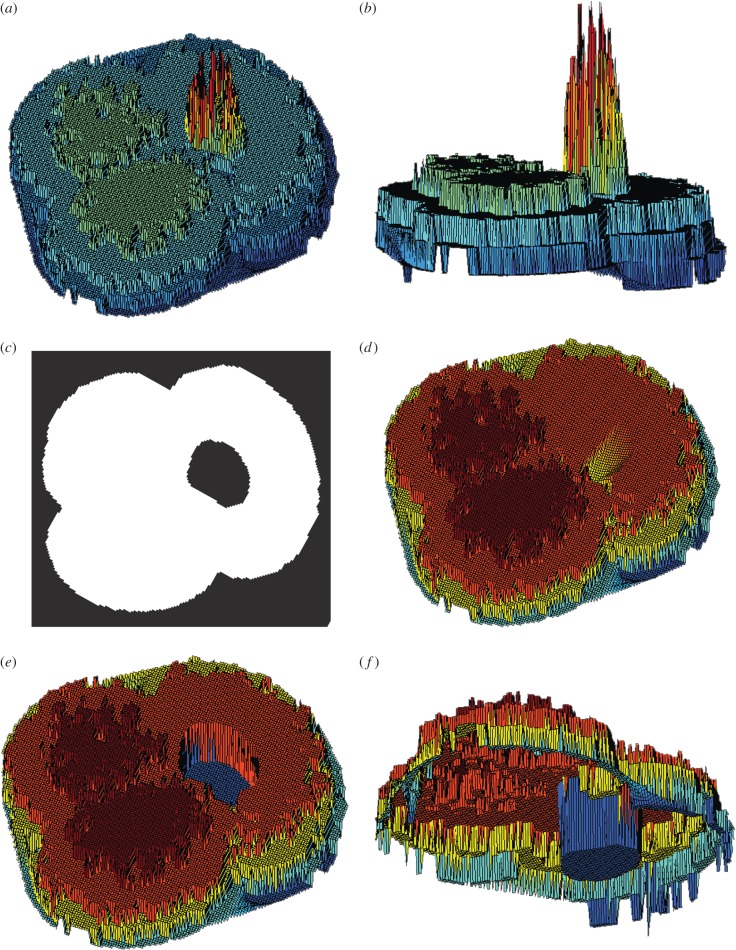
Correction of artefacts arising from foraminifer apertures during 3D mesh extraction. (*a*) Example of aperture artefact, which manifests as a large peak of noise. (*b*) Lateral view of the aperture artefact. (*c*) Binary outline of the object with aperture excluded, as outputted by an adjusted version of the *run2dmorph* software. This binary image is then used, via element-wise multiplication, to remove the aperture during initial mesh extraction (*d*). The aperture height is then artificially set to the lowest height value in the mesh, as illustrated in top (*e*) and bottom view (*f*).

More specifically, the semi-3D mesh extraction pipeline is as follows: First, all images are rescaled such that pixels are squared and 1 pixel = 1 µm (though any base unit can be used), using the conversion factor generated by the microscope calibration (0.975 µm/pixel for both the *x*- and *y*-dimensions in all cases here). The 2D outline from the *run2dmorph* module of the *AutoMorph* software package [55] is then used to exclude the background of the height map (figure 2*b*). This effectively deletes all background noise before the 3D mesh extraction step (figure 2*c*). The presence of a pronounced aperture (the opening in the final chamber) in some foraminiferal species resulted in errors in 3D mesh extraction owing to *Stack Focuser*'s inability to capture aperture depth with fidelity. In most cases, pronounced apertures resulted in large noisy spikes in the final 3D mesh (figure 3*a*,*b*). To mask apertures, *run3dmorph* calls and runs an adjusted version of *run2dmorph* that skips the hole-filling step (figure 3*c*), thus allowing the aperture to be identified and excluded along with the background (figure 3*d*–*f*).

The cleaned height map (figure 2*c*) is then used to extract the height of each pixel based on the value of each pixel (between 0 and 255) in the height map, the number of slices in each *z*-stack (in our case, 31 *z*-slices) and the distance between each *z*-stack slice (31.1 µm), where the extracted height of each pixel was equal to:
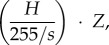
where *H* is the greyscale value of a pixel in the height map, *s* is the number of slices in the *z*-stack for the object in question, and *Z* is the distance between each slice in µm. This results in a matrix *A* with dimensions *x* × *y*, where *x* is equal to the width of the object and *y* is equal to the height of the object, and where each entry *a_ij_* corresponds to the actual height, in µm, of the pixel located at *i*,*j*. The accuracy of this approach in extracting heights from planktonic foraminifera was checked using the spherical species *Orbulina universa*. For the seven specimens examined, the extracted height was within 7.67% of the major and minor axes length.

A semi-3D mesh is then extracted from the scaled height map whereby every non-zero pixel of the height map is given a point in *x, y, z* coordinate space (i.e. *x* = horizontal, *y* = vertical, *z* = height). The extracted 3D mesh is then passed through a custom sliding neighborhood filter to remove outlier noise (figure 2*d*). This filter processes a kernel of size *n* × *n* pixels (where *n* is an odd positive integer). For each pixel, the filter calculates the upper and lower quartile ranges of all the pixel values encompassed in the neighbourhood (i.e. the *n* × *n* kernel). If the focal pixel falls outside the inner quartile range (i.e. below 25% or above 75%), it is considered an outlier and replaced with the mean value of all the pixels in the neighbourhood. This filter has the effect of removing both high and low outliers that result from noise created during height map generation, and also of smoothing the surface of the final mesh. For our specimens, we used *n* = 45 after testing several values of *n* to optimize processing time, noise deletion effectiveness and smoothing. Larger values of *n* are required to effectively delete larger patches of noise, but result in significantly increased computational resource requirements, and may also result in over-smoothing of the final mesh. The optimal balance between the kernel size used for height map generation and the kernel size used for outlier filtering varies between objects/sample sets, and must be determined through testing for each particular dataset.

Once the height map is filtered, the number of pixels present on each *z*-level (i.e. height) is counted. If the number of pixels in a given *z*-level is smaller than 1% of the total number of pixels in the object, that *z*-level is removed. This step has the effect of deleting any background noise along the edge of the object that may have been missed by the previous outlier filtering step. For our foraminifera, we also removed the bottom-most *z*-level for every object, as the majority of the meshes retained a rim of background around the object, thus obscuring the outline of the shape. Finally, all objects with apertures are then given an aperture depth equal to the lowest height in the overall object (figure 3*f*). Because the semi-3D meshes consist only of the visible upper portion of the foraminifera, this method results in apertures terminating approximately in the centre of the foraminifera.

Once fully processed, the semi-3D mesh is then extracted as a series of vertices and faces using the *pointCloud2mesh* function from the *geom3d* package [85] and saved in both Wavefront OBJ and Object File Format (OFF) format. The 3D mesh can be downsampled when saving (to minimize file sizes) but, for our purposes, the full mesh was retained (i.e. no downsampling was conducted). A CSV file containing the raw *x*-, *y*- and *z*-coordinates is also saved at this step. For quality control, *run3dmorph* can also output 3D PDFs of the extracted meshes, and uses the *u3d_pre* [87] function, a modified version of *save_idtf*, *IDTFConverter* (all from the *mesh2pdf* package; [88,89]) and the *LaTeX* package *media9* (v.0.60; [90]) to do so.

**Figure 4.**
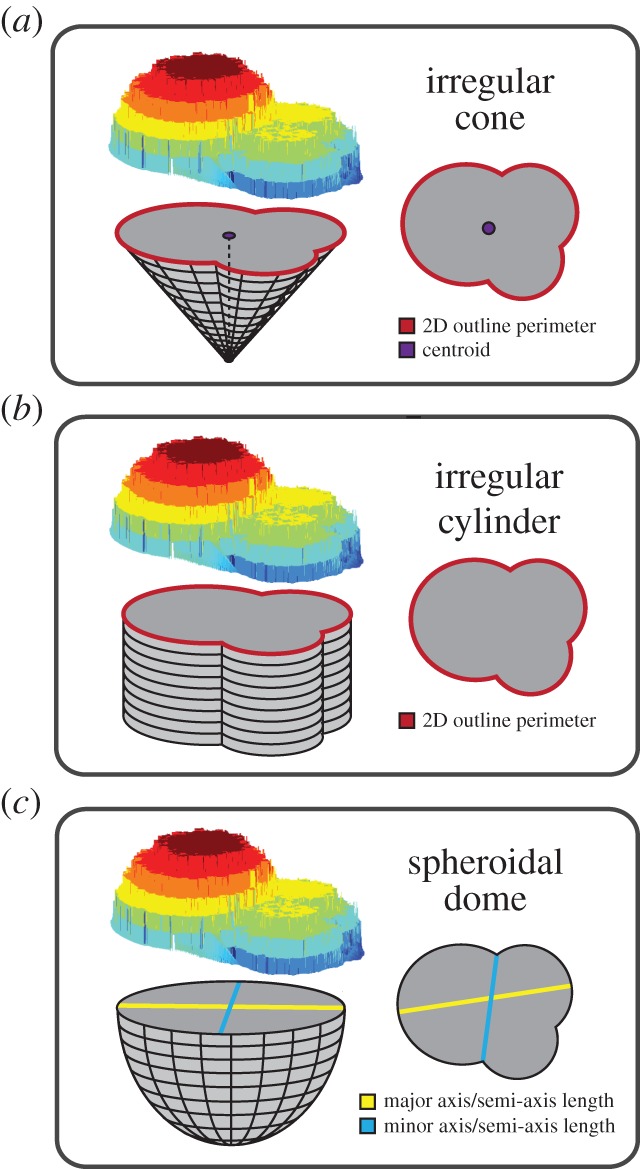
Illustration of idealized base shapes, used by *run3dmorph* for estimating surface area and volume of the complete object. (*a*) Irregular cone base; (*b*) irregular cylinder base; (*c*) spheroidal dome base.

In addition to semi-3D meshes, *run3dmorph* also estimates the volume and surface area of all objects, and saves an additional CSV file of these values. The volume and surface area of the extracted semi-3D hull are calculated exactly (by summing up the heights (or areas) of every pixel), while the volume and surface area of the bottom, un-imaged half are estimated. Three estimations of the bottom half shape are made in order to bound the volumetric and surface area uncertainty arising from the lack of direct measurement. They include base shapes of an irregular cone (figure 4*a*), an irregular cylinder (figure 4*b*) or a spheroidal dome (figure 4*c*). The surface area and volume of the irregular cylinder are calculated as *P*_2D_ × *H* + *A*_2D_ and *A*_2D_ × *H*, respectively, where *P*_2D_ = the length of the perimeter of the 2D outline, *H* = height and *A*_2D_ = the area enclosed in the 2D outline. Height (*H*) above the background is calculated as the distance between the lowest image plane (e.g. the slide background) and the lowest *z*-level of the extracted semi-3D mesh. The 2D parameters, perimeter length and 2D area are outputs of *run2dmorph*.

The volume of the spheroidal dome is calculated as 1/2 * 3/4 * *πs_x_s_y_s_z_*, or one-half of an ellipsoid where *s_x_* = semi-axis *X*, *s_y_* = semi-axis *Y* and *s_z_* = semi-axis *Z*. For the dome, semi-axis *X* is equal to the length of the major axis of the 2D outline and semi-axis *Y* is equal to the length the minor axis of the 2D outline (figure 4*c*). Semi-axis *Z* is equal to the height *H*. The surface area of the spheroidal dome is estimated using Thomsen's formula, where *k* = 1.6:
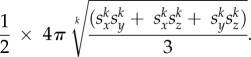


Although the volume of the irregular cone can be calculated as one-third of the volume of the irregular cylinder with the same base, the surface area of the irregular cone cannot be calculated exactly. We estimate the surface area of the irregular conical surface using 100 perimeter coordinates extracted from the 2D outline, the height (*H*) and centroid of the cone, and the angle of inclination from each perimeter point and the centroid. The centroid of the 2D outline is determined using the *MATLAB regionprops* function, and then the angle of inclination (*φ*) between the horizontal and the line formed between the centroid and each perimeter coordinate is calculated, such that *φ* increases monotonically along the perimeter from 0 to 2*π*. The corresponding Euclidean distance between each perimeter coordinate and the centroid is also calculated. Then, using the midpoint integration rule, the generalized conical surface area is estimated by summing the areas of the trapeziums formed between each adjacent perimeter coordinate and a point of height *H* directly above the centroid.

The bottom estimates (conical, cylindrical and spheroidal) are used because they represent the full range of possible background shapes—from perfectly domed in the spherical *Orbulina universa*, to conical in *H. hirsuta,* to flat or filling-in taxa like *T. truncatulinoides and N. dutertrei*. They also span the theoretical maximum (cylindrical) and minimum (conical) possible volumes (or surface areas) of the back-half and thus allow for estimating the range of possible volumetric uncertainty introduced by assuming the shape of the back-half of the object. This uncertainty is calculated as
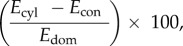
where *E*_con_ = estimate of volume (or surface area) of the object with a conical back, *E*_cyl_ = estimate of volume (or surface area) of the object with a cylindrical back and *E*_dom_ = estimate of volume (or surface area) of the object with a spheroidal back.

### (e) Mesh alignment and landmark placement for semi- and full-3D morphometrics

**Figure 5.**
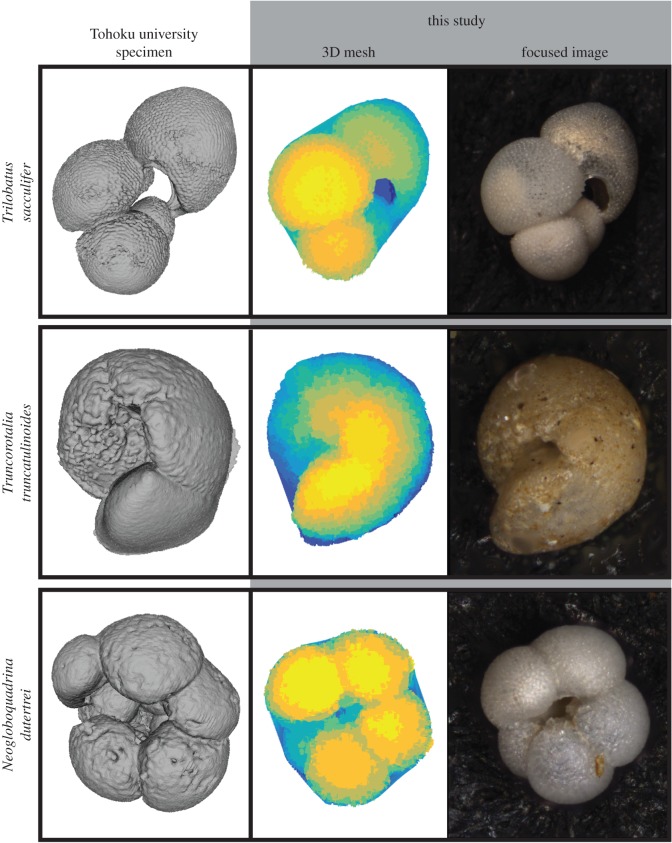
Visual comparison of data types: Tohoku University 3D specimens from CT and semi-3D half-hulls extracted using the *run3dmorph* software on stacked microscopic images. Three examples specimens are shown: *Trilobatus sacculifer*, *Truncorotalia truncatulinoides* and *Neogloboquadrina dutertrei*. The Tohoku University specimens were digitized using X-ray CT at 5 µm resolution. *Run3dmorph*-extracted 3D-meshes are shown next to their corresponding focused 2D-image.

In this study, we use 3D semi-landmark geometric morphometrics to assess the morphology of species and communities. To use these analytical approaches, we first had to convert the semi- and full-3D meshes (from our specimens and the Tohoku University specimens) to landmarks. For a visual comparison of these two data types, see figure 5. Landmarks were placed using Boyer *et al.*'s [91] automated alignment and shape comparison algorithm, as implemented in the *R* (v. 3.0.2; [92]) package *auto3dgm* [93] and the parallelized cluster version of the algorithm implemented in *MATLAB* as *PuenteAlignment* [94]. Analyses using *auto3dgm* were conducted on the Tide server, whereas analyses using *PuenteAlignment* were conducted on the Yale High Performance Computing Omega cluster.

Two batches of landmark placement analyses were run for the semi- and full-3D specimens respectively. For the semi-3D specimens, landmarks were optimized using the parallelized *PuenteAlignment* for 597 objects that comprise the 281 species-identified individuals from the five Atlantic coretops and selected exemplar species, the 200 reproducibility tests ‘individuals’, and an additional 116 complete and well-imaged individuals (without species-level identification) from the five Atlantic coretops, examined by eye to ensure proper mesh extraction. For the full-3D analysis, landmarks were optimized using *auto3dgm* for the 39 Tohoku University specimens. For both the semi- and full-3D analyses, 256 landmarks per object were placed and used in the subsequent construction of morphospaces.

### (f) Morphospace construction

Four morphospaces were constructed to consider the key questions of the study: What is the relationship between morphology, ecology and evolutionary history in extant planktonic foraminifera? And, can semi-3D morphometrics capture community dynamics? To get at these issues, we first constructed two morphospaces for the complete set of semi- and full-3D data, respectively (comprising a total of 587 semi-3D individuals and 39 full-3D individuals). In order to directly compare morphospaces between the semi- and full-3D approaches, we also constructed two morphospaces, one for semi-3D and one for full-3D, including only the 16 overlapping species between the two datasets (e.g. the 15 macroperforate species listed in table 1 and the microperforate *C. nitida*). The pruned semi- and full-3D datasets contained 421 and 34 individuals, respectively.

In each case, morphospace was constructed using the *Geomorph R* package [95]. We first used the *gpagen* function to conduct a Generalized Procrustes Alignment (GPA) of the *auto3dgm-*/*PuenteAlignment-*extracted landmarks, with all landmarks set as sliding surface semi-landmarks. The reason for using semi-landmarks is twofold: first, as the Boyer *et al.* algorithm does not assume homology between landmarks [91], the resulting landmarks are not true geometric morphometric landmarks and should be allowed to move to minimize the potential distance between objects in morphospace. Second, Gonzalez *et al.* [96] found curvature in shape space when using the landmarks outputted by the Boyer *et al.* [91] algorithm as traditional landmarks, a pattern that we also observed in the planktonic foraminiferal shape spaces. With semi-landmarks, shape space is uncurved, supporting the use of semi-landmarks for a more accurate characterization of shape space. After a GPA is conducted on the semi-landmarks, a principal component analysis (PCA) was then conducted using the *plotTangentSpace* function in the *Geomorph* package. The resulting principal components capture the major axes of variation in the full- and semi-3D morphospaces for planktonic foraminifera.

### (g) Hierarchical clustering: morphology and ecology

Relationships among planktonic foraminifera in shape space (and subsequently, ecological space) were considered using hierarchical clustering with the *hclust* function in *R*. Hierarchical clustering was carried out on the complete full- and semi-3D morphological datasets (containing 39 and 587 individuals, respectively), and on the pruned datasets containing only the 16 species present across both datasets (table 1; containing 34 and 421 individuals, respectively).

For the morphological clustering, principal component (PC) scores were first averaged within species along each PC. A Euclidean distance matrix was then calculated for the species-averaged PC scores. This distance matrix was then used for hierarchical clustering using the Ward, single, complete, average and McQuitty linkage agglomeration methods in *hclust*. A 50% majority-rule consensus tree was then built from the resulting dendrograms using the *consensus* function from the *ape* (v. 3.0-10; [97]) *R* package in order to identify stable clusters. The final consensus dendrogram depicts consensus linkages between each species in the various analyses.

For the ecological clustering, ecological characteristics (symbiont type, habitat depth and geographical range) were taken from Ezard *et al.* [98] for 15 of the 16 overlapping species (*C. nitida*, the only non-macroperforate species, was excluded). Jaccard distances were then calculated between each species using the *vegdist* method from the *vegan* (v. 2.2-1; [99]) *R* package. Using the Jaccard distance matrix, hierarchical clustering and consensus dendrogram generation were conducted as described above.

### (h) Linear discriminant analysis for semi-3D data

One of our key findings (discussed in detail below) is that species overlap to a much greater degree in semi-3D morphospace than in full-3D morphospace. Reasoning that the species, identified by PMH based on morphology, are, in fact, morphologically distinct, we conducted a linear discriminant analysis (LDA) on the principal component coordinates prior to conducting hierarchical clustering for the semi-3D dataset. This was done to bring the variance that is useful in distinguishing species clusters to the forefront of the semi-3D analysis, thereby minimizing the potential noise introduced by the current limitations of the semi-3D approach. We constructed the PCA–LDA model using the function *lda* from the MASS package [100], with PCs 1 through 457 (the maximum number of PCs that could be included without resulting in collinearity) as the continuous explanatory variables and species identity as the dependent categorical variable. The *predict* function was then used to apply the linear function derived from the LDA for hierarchical clustering and, later, for the examination of morphospace occupation by the five North Atlantic sites. Clustering on LDA output followed the procedure described above for the PCA data.

### (i) Assessing topological similarity: morphology, ecology and phylogeny

An analysis of topological similarity was used to assess the overall similarity of morphospace as captured by the semi- and full-3D analyses, and to examine the relationship between planktonic foraminiferal morphology, ecology and phylogeny. These analyses were conducted using the consensus dendrograms for the semi- and full-3D morphological clustering, the ecological clustering, and a pruned version of Aze *et al*.’s [22] phylogeny of macroperforate foraminifera pruned to the 15 macroperforate species common to all datasets (table 1). Topological similarity was assessed by calculating the path difference (PD) [101] between unrooted topologies using the *treedist* function from the *phangorn* (v. 1.99-1) [102] *R* package. Because the path difference is only well defined for fully bifurcating trees, we randomly resolved multifurcations using the *multi2di* function from the *ape R* package. To account for differences arising in tree topology as a result of randomized uncertainty resolution, we conducted 2000 replicates for each dendrogram that required multifurcation resolution and report the average path difference of all replicates.

### (j) Assessing phylogenetic signal in ecology and morphology

Phylogenetic niche conservatism describes the pattern of closely related species exhibiting similar ecological traits [103]. To assess the degree of niche conservatism present in our ecological dataset, we calculated Pagel's *λ* using the time-calibrated Aze *et al.* [22] phylogeny pruned to include only the 15 macroperforate species that overlap in the Tohoku University and semi-3D datasets (electronic supplementary material, figure S1). These pruned datasets contained 33 full-3D individuals and 414 semi-3D individuals. The Aze *et al.* [22] time-calibrated phylogeny was built using the data for a fully bifurcating morphospecies tree in the *R* package *paleoPhylo* (v. 1.0-108) [104] and pruned using the *drop.tip* function from the *ape R* package. Pagel's *λ* was then calculated for each of the three ecological characters (i.e. symbiont type, habitat depth and geographical range) using the *phylosig* function from the *phytools R* package (v. 0.4-31) [105].

Phylogenetic signal was also assessed using Pagel's *λ* for each individual principal component as a measure of the degree of phylogenetic signal present in the morphological data, and as a proxy for identifying which PCs are most informative in both the full- and the semi-3D datasets. For every PC (39 total for the full-3D Tohoku University dataset and 597 for the semi-3D dataset), Pagel's *λ* was calculated using the average PC values for each of the 15 focal macroperforate species as described above (results in the electronic supplementary material, table S2). The morphological PCs with high phylogenetic signal and strong support (i.e. *λ* > 0.5 and *p* < 0.05) were identified for each dataset (electronic supplementary material, table 2*a*,*d*). These high phylogenetic signal PCs are interpreted to capture the phylogenetically informative aspects of morphology. To understand more specifically what aspect of shape variation these phylogenetically informative orthogonal axes were capturing, the five maximum and minimum individuals along the top three high-*λ* PC axes (PCs 2, 4 and 12 for the Tohoku University dataset, and PCs 2, 17 and 96 for the semi-3D dataset) were identified and considered in turn (electronic supplementary material, table S2*b*,*e*).

**Table 2.** Comparing topologies of morphological, ecological and phylogenetic clusterings using the path difference pairwise distance metric. Lower distances correspond to more similar topologies.

	path difference			
	Tohoku University	coretop/exemplar	ecology	phylogeny
Tohoku University	—			
coretop/exemplar	32.882	—		
ecology	35.642	24.570	—	
phylogeny	32.019	24.429	23.367	—

For each morphological dataset, hierarchical clustering was then conducted for: (i) all of the PCs with high phylogenetic signal and strong support and (ii) the PC with the highest *λ* value and a substantial amount of morphological variance captured (cutoff of 3% or more) (i.e. PC 4 for the Tohoku University dataset and PC 2 for the semi-3D dataset). The path difference between the high-*λ* PC dendrogram, the single highest PC dendrogram, and the Aze *et al.* [22] phylogeny was then calculated for each dataset (electronic supplementary material, table S2*c*,*f*). Hierarchical clustering and tree distance calculation was carried out using the same methods described in §2g,i.

## 3. Results

### (a) Pipeline for semi-3D morphometrics: towards high-throughput community dynamics

With the completion of the beta version of *run3dmorph*, we have a complete pipeline for the extraction of semi-3D morphometric data (including surface area and volume estimates) from light microscopic and photogrammetric images. The first two components of the pipeline (*segment* and *focus*) are written in Python, a free programming language that runs across platforms. *run2dmorph* and *run3dmorph* currently execute in *MATLAB* (version 2015b or above), a proprietary software, but will be ported into Python in future versions. All the software is available, with frequent updates, from GitHub (see https://github.com/Hull-Lab). Key features of *run3dmorph*, discussed in detail in Material and methods, are aggressive noise reduction routines (figures 2 and 3) and multiple options for estimating surface area and volume given unknown backs to objects (figure 4). From the angle imaged, semi-3D meshes visually reflect the 3D morphology captured by more traditional, CT scanning methods (figure 5).

### (b) Semi- and full-3D morphospace in modern planktonic foraminifera

#### (i) Tohoku University specimens and full-3D morphospace

**Figure 6.**
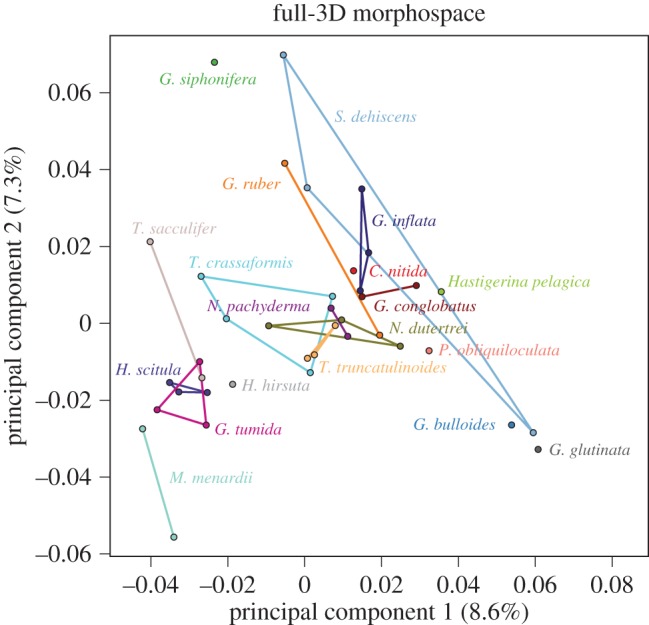
Morphospace (PC1 versus PC2) generated from the 39 specimens that comprise the Tohoku University dataset, aligned using the Boyer *et al.* automatic 3D geometric morphometrics algorithm [91] and sliding semi-landmarks. Coloured lines define convex hulls for each species (for species that include only two individuals, this is manifested as a single connecting line). *H. pelagica*, *Hastigerina pelagica*; *H. scitula*, *Hirsutella scitula*; *G. bulloides*, *Globigerina bulloides*; *G. glutinata*, *Globigerinita glutinata*.

We constructed the first 3D morphospace for modern planktonic foraminifera using the CT scans of 39 Tohoku University specimens spanning 19 extant taxa (figure 6). Across all of the datasets that we examined, we found that the morphological variation captured by single principal component axes was always quite low: in the case of the full-3D morphospace, the first three PCs cumulatively account for just 21.24% of the total morphological variance, with PC1 accounting for 8.61%, PC2 for 7.32% and PC3 for 5.31%. However, we do not find this low variance captured inherently problematic. In allowing the semi-landmarks to slide, Gonzalez *et al.* [96] found that the amount of variance capture in the first two principal components fell by about half for rodent molars, rodent brains and primate brains, with sliding 3D-semi-landmark PC1 values ranging from 18–40% of total morphological variance. We likewise found a similar decline in variance capture between non-sliding and sliding semi-landmarks of about half. Even so, the variance captured in these 3D measures of planktonic foraminiferal shape seem low considering that 2D outline methods can capture approximately 75% of morphological variance in the first three principal components (i.e. [19]). Although we have yet to compare 2D and 3D morphometrics in planktonic foraminifera, we suspect that the drop in variance captured is owing to the fact that much of the shape variation in the third dimension is independent of that in the other two dimensions. This remains to be tested in future work.

The low morphological variance captured by the first few PCs does not affect the robustness of the exploratory morphological analyses, as these are conducted using all non-collinear PCs (i.e. excluding highly correlated PCs). The non-collinear PCs together capture more than 95% of the total variance.

**Figure 7.**
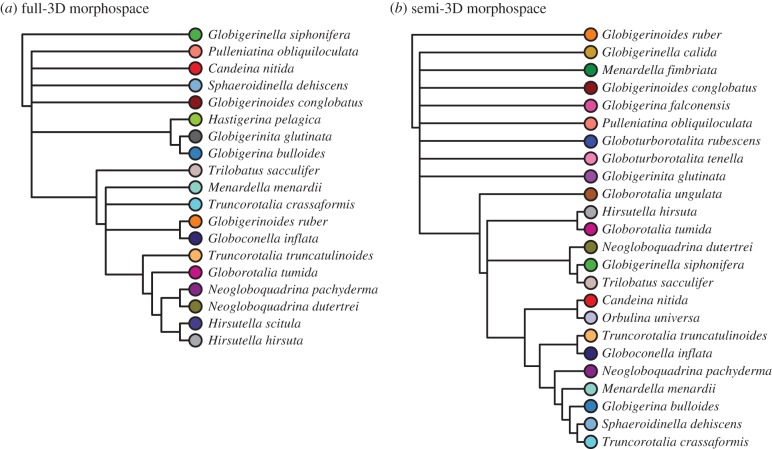
Majority-rule consensus cluster dendrograms for (*a*) the Tohoku University dataset and (*b*) our novel coretop exemplar dataset. Tip label colours correspond with species colours in the morphospaces depicted in figures 6 and 8*a*. The consensus dendrogram is constructed from five cluster dendrograms, each built from the same mean distance matrix using a different clustering algorithm (see text).

For the Tohoku University specimens, overlap in the relative position of species in morphospace generally occurred between taxa with close evolutionary affinities. For instance, in PC1/PC2 space (figure 6), the two *Neogloboquadrina* species overlap (*N. dutertrei* and *N. pachyderma*) and the two *Truncorotalia* species overlap (*T. crassaformis* and *T. truncatulinoides*) and all the disc-shaped taxa (technically ‘globorotaliform’) cluster in the lower left quadrant of morphospace (e.g. *Menardella menardii*, *H. hirsuta*, *Hirsutella scitula* and *Globorotalia tumida*). The morphological clustering of some taxa by taxonomic affinity is also clearly apparent in the consensus dendrogram (figure 7*a*). Compact to spherical forms, spanning a range of taxonomic groups, occupy the centre of PC1/PC2 space and include the closely related *Globigerinoides ruber* and *Globigerinoides conglobatus*, along with other taxa (e.g. *S. dehiscens*, *Globoconella inflata*, *P. obliquiloculata* and the Neogloboquardiniids). More lobulate forms (e.g. *Globigerina bulloides* and *Globigerinita glutinata*) have the most positive PC1 scores. The largest amount of morphological variation along PC1–PC2 is encompassed by *S. dehiscens*, a species noted for its unusual crust with supplementary apertures. The next largest amount of variation is encompassed by *T. crassaformis*, which exhibits a cone-like axial form that varies greatly within the pseudeo-cryptic species complex [53].

When the phylogenetic signal of each individual PC in the full-3D dataset is assessed using Pagel's *λ*, the PCs with the strongest phylogenetic signal are PC2, 4, 12 and 39 (electronic supplementary material, table S2*a*). Although PCs 2 and 4 have a strong phylogenetic signal (*λ* of 0.66 and 0.82), the species representing the extremes of the PC axes do not sort out according to species or morphotype (electronic supplementary material, table S2). For instance, *S. dehiscens* appears as a representative species on both ends of the PC2 axis.

#### (ii) Semi-3D morphospace

**Figure 8.**
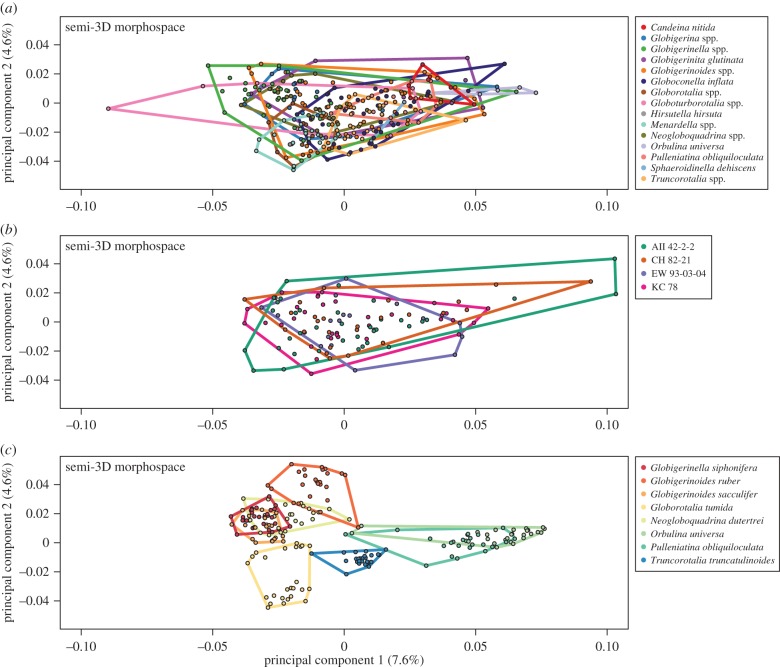
Morphospace generated from the 597 specimens that comprise our novel coretop/exemplar dataset, aligned using the Boyer *et al.* automatic 3D geometric morphometrics algorithm [91]. (*a*) The 281 species-identified coretop/exemplar individuals, grouped by genus to aid visualization (note: *Trilobatus sacculifer* grouped with *Globigerinoides*); (*b*) the 116 individuals from four Atlantic coretops; (*c*) the 200 reproducibility test individuals. Coloured lines define convex hulls for each genus/coretop/species. All three morphospaces are plotted on the same axes (PC1 versus PC2) and scale.

The 281 individuals from 24 species, the 200 replicate ‘individuals’, and the 116 coretop objects were used to construct the first semi-3D morphospace for modern planktonic foraminifera. As with the full-3D morphospace, the variance captured by the first three PCs was low (14.66% total), with PC1 accounting for 7.6%, PC2 for 4.6% and PC3 for 2.5% of the total variance. A striking difference between the semi- and full-3D morphospaces is the amount of overlap between different species (e.g. figure 8*a*) along all PCs. The low number of individuals included in the full-3D analyses precludes a quantitative analysis of the relative overlap between species in the full- and semi-3D approaches, but it is clear that while the full-3D analysis resulted in sensible separation of major taxonomic groupings along the principal PCs, the semi-3D analyses did not.

This complete overlap in semi-3D space is also apparent in the complete morphological overlap between North Atlantic sites with very different species compositions (figure 8*b*), and in the wide variance observed when the same individual is imaged under the multiple reproducibility test conditions, as described in §2b (figure 8*c*). This latter analysis (figure 8*c*: repeat measurements on single individuals) reveals the general tendencies of PC1 and PC2 in semi-3D morphospace, with a progression from relatively low (e.g. flat) to high (e.g. domed) umbilical profiles on PC1 and from smooth-edged to lobulated-edged along PC2. Based on Pagel's *λ*, PC2 is identified to be one of the most taxonomically informative PCs for the semi-3D dataset, along with PC 17, 96, 457, 555 and 588. As with the full-3D data, the taxa loading on the extremes of these PCs are typically mixed (e.g. *Globigerinoides ruber* appears at both extremes of PC17), with the exception of PC2. For PC2, five individuals of *G. ruber* have the most positive PC2 scores, and five individuals of *Menardella menardii* and *Globorotalia tumida* have the lowest scores.

**Figure 9.**
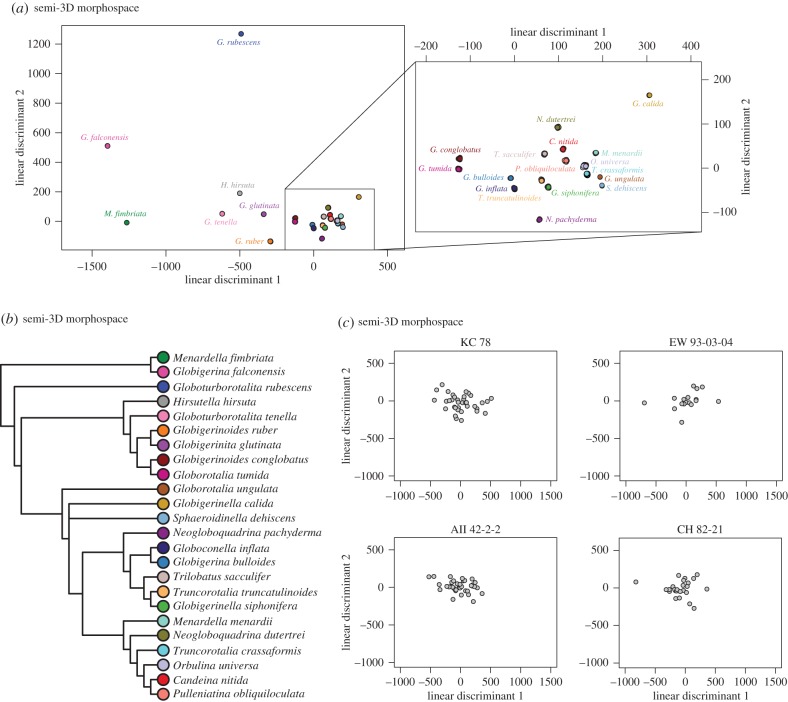
(*a*) Plot of the linear discriminant (LD) space (LD1 versus LD2) for the 24 planktonic foraminifer species present in the semi-3D dataset. Zoomed-in box shows the enclosed species on different axes to show relative cluster positions more clearly. (*b*) Corresponding majority-rule consensus cluster dendrogram for the LD space shown in (*a*). (*c*) LD spaces for coretop individuals, with species identity predicted using the LD model estimated using the species-identified individuals in (*a*) as the training set. Each coretop is plotted with identical axes to show relative occupation.

**Figure 10.**
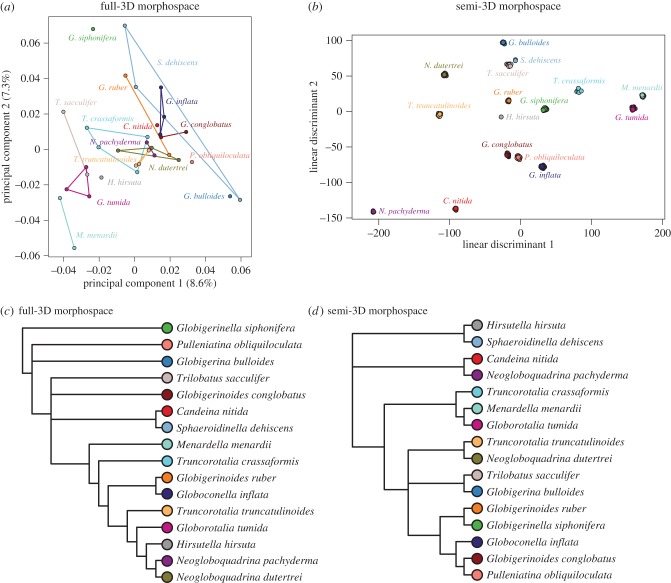
Morphospaces and majority-rule consensus cluster dendrograms for the 16 species that overlap between the Tohoku University dataset (*a*,*c*) and our novel coretop/exemplar dataset (*b*,*d*) (post-LDA; see text). Coloured lines define convex hulls for each species. Dendrogram tip label colours correspond to morphospace species colours.

The high overlap between species along all the PCs directly examined led us to perform a PCA–LDA on the semi-3D data before clustering. The PCA–LDA allowed us to examine the relationships among taxa using the variance relevant to distinguishing among species (cf. figure 7*b* (PCA cluster) and figure 9*b* (PCA–LDA cluster)). To consider the relationship between the semi- and full-3D datasets, we performed a second PCA–LDA that included just those 16 species overlapping between the semi- and full-3D datasets (figure 10). This second PCA–LDA emphasizes the difference in morphospace between the full- and semi-3D analyses. There are no species pairs in common between the two analyses (figure 10). Both dendrograms contain a major cluster of nine species, but only four species are in common to both clusters—a result which might be expected by chance.

#### (iii) Semi-3D morphospace: reproducibility test

**Figure 11.**
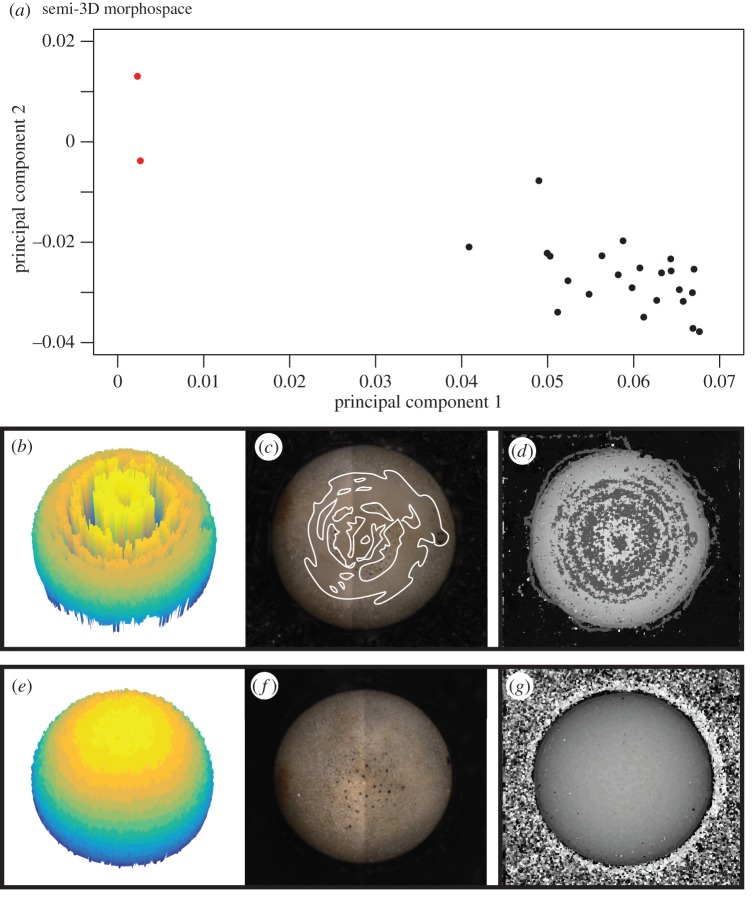
Exploration of the reproducibility tests of the *Orbulina universa* individual (*a*) The PC1 versus PC2 coordinates for *O. universa* from figure 8*c*. The points highlighted in red are outliers for which the *run3dmorph*-extracted semi-3D half-hulls exhibited pathologies (see text). (*b*–*d*) Extracted mesh, focused image and height map showcasing pathologies arising during *z*-stack focusing. In (*c*), the smeared, unfocused portions of the object are outlined in white. (*e*–*g*) The corresponding mesh, focused image and height map for a properly extracted *O. orbulina* individual.

To explore the question of ‘other sources of variance’, we consider the results of the reproducibility tests in more detail here. For the reproducibility tests, eight individuals (representing eight different species) were imaged under 25 different conditions each, for a total of 200 ‘individuals’ imaged. The goal of this test was to determine how much of the semi-3D morphological variability could reflect various sources of error introduced during slide preparation and imaging, including variable angles of imaging, image compositing and glare. Ideally, the reproducibility test individuals would cluster closely together in morphospace—while this is somewhat true (figure 8*c*), together these eight individuals span most of PC1 and PC2 morphospace. Several individuals exhibit particularly high levels of variation: *N. dutertrei*, *Globigerinoides ruber* and *Orbulina universa* (figure 8*c*). We considered the variability of the perfectly spherical *O. universa* in greater detail (figure 11*a*), and it was apparent that the large variation exhibited by *O. universa* is due in large part to two outlier individuals (marked in red). Examination of the meshes of these two outlier individuals reveals obvious errors in the integrity of the extracted mesh (figure 11*b*), which appear to have resulted from imaging artefacts during *z*-stack focusing (figure 11*c*). These artefacts appear as a smeared, unfocused area on the object surface in the focused image. The error is then perpetuated through the mesh extraction pipeline via a poorly constructed height map (figure 11*d*). The pathological nature of the two outliers is clearly distinguishable via comparison with non-pathological individuals (figure 11*e*–*g*).

Without the two poorly extracted individuals, the variation encompassed by the *O. universa* is comparable to that of the relatively low variation individuals of *T. truncatulinoides*, *Trilobatus sacculifer* and *Globigerinella siphonifera* (figure 8*c*). All of these low variation individuals have relatively flat spiral surfaces and, in two cases, small (*T. truncatulinoides*) or edge-facing (*G. siphonifera*) apertures—both factors that increase the consistency of the image perspective. *Globigerinoides ruber*, *N. dutertrei* and *Globorotalia tumida* have some combination of large apertures and domed-spiral sides, both of which dramatically affect the shape of the semi-3D mesh extracted from slightly different viewpoints.

#### (iv) Full- versus semi-3D morphospace

To facilitate the direct comparison of the full- and semi-3D morphospaces, we trimmed the two datasets down to include just the 16 species in common to both datasets. These 16 species versions were clustered, as before, on the PCA data for the full-3D dataset and on the PCA–LDA data for the semi-3D dataset (figure 10). The most readily apparent feature of the morphological consensus for both datasets is how little the clustering appears to relate to taxonomic affinities. There are, of course, exceptions, with *N. dutertrei* and *Neogloboquadrina pachyderma* paired in the full-3D analysis, and *Menardella menardii* and *Globorotalia tumida* paired in the semi-3D analysis. Beyond this, both trees have structure that can be interpreted as reasonable given the morphology of the taxa in related clusters, but overall the groupings were unexpected given our morphological understanding of these species.

### (c) Relationship between gross morphology, ecology and phylogeny in 15 extant species

The PD tree distance metric was used to quantitatively assess the similarity between the two morphological dendrograms (semi- and full-3D) and to examine the relationship between 3D morphology and the phylogenetic and ecological relatedness of taxa (table 2). As a Euclidean distance-based metric of tree similarity, each tree is represented as a vector of pairwise edge distances between all terminal taxa pairs and is thus compared. This comparison is purely topological (i.e. edge lengths are not accounted for) and provides a direct metric for assessing the topological similarities between the morphological, ecological and phylogenic dendrograms.

The two most similar dendrograms were the ecological and phylogenetic trees (table 2). This result suggested a strong signal of phylogenetic niche conservatism, a possibility we tested further using Pagel's *λ* on the three ecological characters (symbiont type, habitat depth and geological range) individually. In the subset of 15 macroperforate species of planktonic foraminifera examined morphologically, we found phylogenetic signal in all ecological traits: symbiont type (*λ* = 1.049; *p* = 0.002), habitat depth (*λ* = 1.106; *p* = 0.002) and geographic range (*λ* = 0.565; *p* = 0.046).

**Figure 12.**
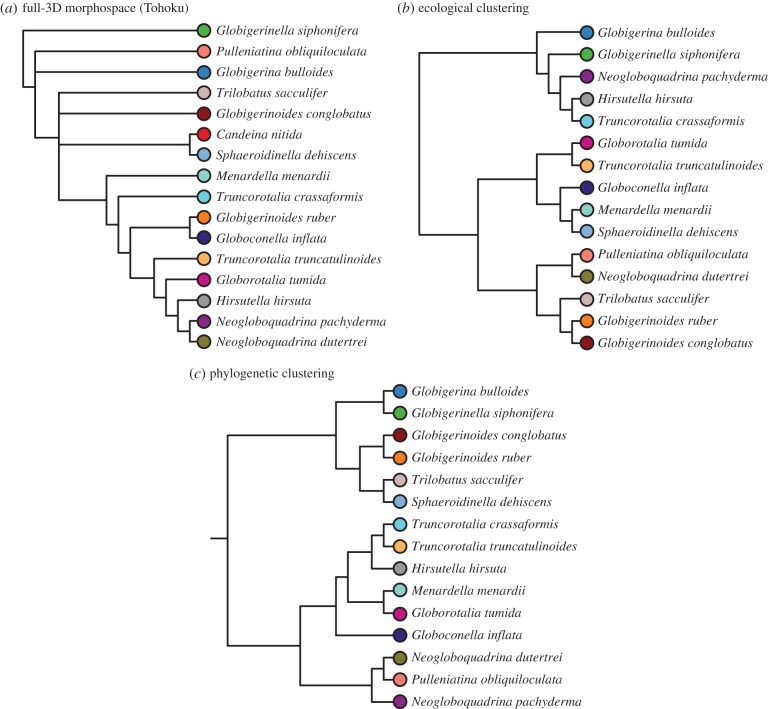
Ecological and phylogenetic clustering of the 15 macroperforate species that overlap between the Tohoku University dataset and the coretop/exemplar dataset. (*a*) Consensus cluster dendrogram of the full-3D Tohoku University specimen morphospace (same as figure 10*c*). (*b*) Ecological cluster dendrogram built using Jaccard distances calculated from three ecological traits (table 1). (*c*) The phylogenetic relationships between the 15 macroperforate species, as pruned and redrawn from Aze *et al.*'s [22] stratophenetic phylogeny. Dendrogram tip label colours correspond to morphospace species colours from figure 9.

Both morphological dendrograms (i.e. full- and semi-3D) are more similar to the phylogeny (i.e. exhibits the shortest PD between the two trees) than they are to one another or to the ecological dendrogram. These results suggest a stronger influence of evolutionary history on morphology than of ecology (figure 12*c*). It also implies that the morphological structures captured by the full and semi-3D approaches differ, given the relative dissimilarity between these two morphological dendrograms. To our surprise, the path difference suggests a greater similarity between the semi-3D dendrogram and phylogeny (PD_semi-3D_ = 23.791) than between the full-3D dendrogram and phylogeny (PD_full-3D_ = 31.081). This same relative ordering is also true of the ecological similarity: there is greater similarity between the ecological dendrogram and the semi-3D morphological dendrogram (PD_semi-3D_ = 24.207) than with the full-3D morphological dendrogram (PD_full-3D_ = 34.728).

The higher congruence between the semi-3D morphological dendrogram and the phylogeny, as compared to the full-3D data, also exists in the morphological dendrograms based on high phylogenetic signal PCs only (*λ* > 0.5 and *p* < 0.05). When the high phylogenetic signal dendrograms are considered (PCs 2, 4, 12 and 39 for the full-3D dataset; PCs 2, 17, 96, 457, 555 and 588 for the semi-3D dataset), the path difference between the semi-3D dendrogram and the phylogeny is lower than that between the full-3D dendrogram and the phylogeny (PD_semi-3D_ = 24.576 versus PD_full-3D_ = 26.609) (electronic supplementary material, table S2*c*,*f*).

### (d) Assemblage-wide ecometrics: community structure in four coretop locations

**Figure 13.**
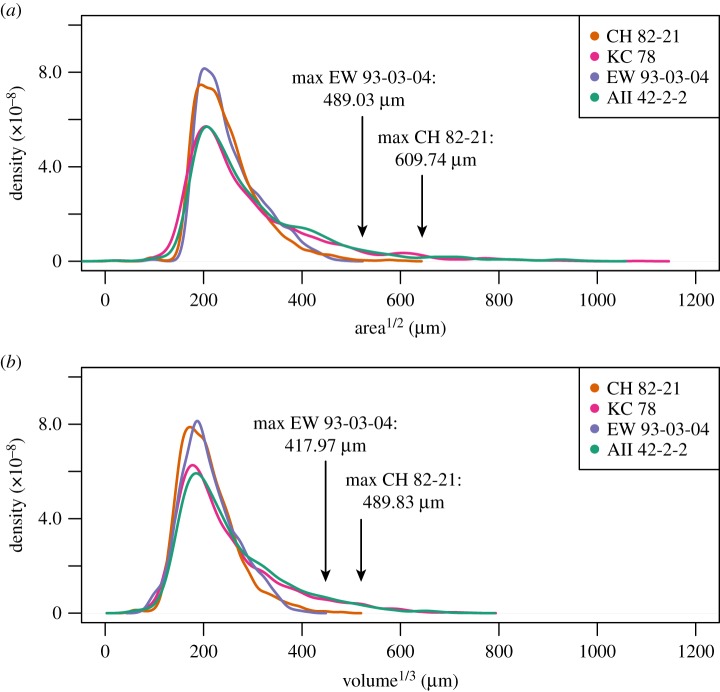
Kernel density plots for (*a*) 2D outline area and (*b*) semi-3D volume (assuming spheroidal dome base) for the four Atlantic coretops: CH 82-21 (*n* = 2879), KC 78 (*n* = 1768), EW 93-03-04 (*n* = 3034) and AII 42-2-2 (*n* = 2000).

In addition to generating 2D-outlines and semi-3D meshes for downstream morphometric analyses, the *run2dmorph* and *run3dmorph* software can also automatically generate estimates of 2D size (e.g. major and minor axes length, enclosed area, perimeter length) and 3D size (volume and surface area). As a key macroecological trait, body size is often measured in fossils either by species exemplars [106,107] or by 2D metrics like major axis length or 2D surface area [108]. For the 9681 individuals included in the community structure analysis from the four coretop locations, the potential uncertainty owing to the unknown back-morphology is estimated as %volumetric difference between the low- and high-end estimates. We find that the high-end estimate (cylindrical) is on average 23.55% (*σ* = 11.89%) larger volumetrically and 3.19% (*σ* = 1.40%) smaller in surface area than the low-end estimate (conical), normalized to the dome estimate. For species like planktonic foraminifera, which range from nearly completely flat to completely spherical, we consider the importance of estimating volume (over 2D area) with a direct comparison of 2D and 3D metrics of community size distributions (figure 13); these comparisons are discussed in the following sections.

## 4. Discussion

In recent years, advances in imaging and image analysis have driven forward our understanding of diversity dynamics in the history of life by unlocking previously inaccessible aspects of the fossil record [109]. There have been three main foci of efforts in this field of virtual palaeontology: (i) the automatic recognition of taxa [110–112], with arguably the most successful application of this approach in calcareous nanofossils [113,114]; (ii) the collection of refined morphometric data [115], with technologically advanced approaches like X-ray and synchrotron CT at the forefront [116,117] and (iii) the rapid assessment of assemblage body size distributions [118], typically in 2D silhouettes [119,120]. High-throughput morphometric approaches have generally lagged because in morphometrics, as in most things, the devil is in the details. Small differences in specimen orientation can introduce more variation into a dataset than evolution itself, and many taxa are differentiated at the species level by fine features missed by conventional methods like light microscopy.

In full recognition of the ultimate need for precision, we set out to develop a high-throughput approach (semi-3D morphometrics). We reasoned that image processing and machine learning will ultimately allow us to extract the ecological and evolutionary signals from the noise and that rapid approaches are critically needed to address questions of assemblage dynamics (see call for population studies in [109,121]). Towards this end, we have developed and tested a method for the extraction of 3D hulls from light microscopic and photogrammetric images, examined the potential of morphology as a tracer of assemblage dynamics in planktonic foraminifera, compared rapid and refined 3D morphometric approaches (e.g. semi- versus full-3D meshes), and explored a preliminary application of these methods in the North Atlantic. We discuss each in turn.

### (a) Towards high-throughput community dynamics

Coupled with upstream functions in the *AutoMorph* software suite, the code presented here (*run3dmorph*) can process a slide scan containing thousands of individual fossils and extract individual semi-3D meshes and volume and surface area estimates for all unique objects (figures 2–5). This code is flexible enough to work with any light-coloured object imaged on a black background, and we are currently using it in-house with photographs of limpet assemblages. All code is freely available on GitHub (https://github.com/HullLab) and is being actively updated to fix known bugs, increase functionality and speed, and remove dependencies on proprietary software. Our reproducibility tests highlight the robustness of the semi-3D mesh extraction code—the two major problems encountered (artefacts during *z*-stack image focusing and/or tilt variation during sample preparation prior to microscope imaging) do not result from the 3D mesh extraction code itself. Rather, the 3D mesh extraction behaves as expected, with the quality of output determined by the quality of the input (an example of mesh extraction resulting from optimal versus suboptimal inputs is shown in figure 11). However, regardless of where the errors occur in the high-throughput approach, they need robust solutions to enable high-throughput study of community dynamics. We discuss ongoing work to address these problems below. At present, in light of the reproducibility test results, users of the *run3dmorph* software are advised to exercise care during initial set-up and positioning of specimens prior to imaging in order to reduce the amount of variability due to tilt, and to inspect 3D PDFs of height map meshes to ensure proper mesh extraction.

Already, *run3dmorph* provides a robust means of measuring assemblage body volume distributions. Even without measuring the backs of objects, we are able to measure the volume of fossils with an uncertainty of approximately 20% introduced by estimating back-half shape. In other words, an individual with a fully filled (i.e. cylindrical) back is approximately 40% more volumetric than the cone-backed equivalent. Although still somewhat uncertain, this is a dramatic improvement on the existing 2D silhouette volume-estimation approaches for assessing assemblage body size distributions, given the great variation among foraminiferal species in the depth dimension. In modern tropical assemblages, some of the largest individuals, as measured by 2D length and width, are also the flattest (i.e. *Menardella menardii*, *Globorotalia tumida*, *H. scitula*, to even relatively flattened taxa like *Trilobatus sacculifer* and *Globigerinella siphonifera*). By assuming that 2D silhouette captures the true body volume, we calculate that 2D methods effectively overestimate the body mass in flat individuals by roughly 375% (as calculated for *Menardella menardii* assuming spherical equivalent). As such, our approach reduces the volumetric uncertainty by more than ninefold, yielding body size measurements that are more accurate and better suited to many of the ecological and morphological questions targeted with body size data. Through its effect on metabolic rates and intraspecific interactions, body mass is considered to be an ecological trait of first-order importance [122,123]. We therefore see the volume (and surface area) estimates of *run3dmorph* as one of the most useful, and certainly most readily applicable, outputs of the code.

Two major advances are planned for the *AutoMorph* software in order to enable accurate 3D reconstructions of assemblages morphometrically by targeting the two major problems in the current analyses: noise and perspective. First, an additional noise reduction step, based on inferred surface rugosity, will be used as a final check for both pitting (as in the *Orbulina* example in figure 11) and glare peaks. Second, we aim to use the semi-3D meshes as hulls for matching, and warping, full-3D shapes to fit. In this way, the semi-3D information can be used to fit and interpret the full 3D-shape of the geometrically formed planktonic foraminifera.

### (b) Determinants of gross morphology in planktonic foraminifera

Planktonic foraminiferal taxa differ in gross and fine morphological features—only the first of which is accounted for here. Fine morphological features beyond our ability to measure include differences in foraminiferal wall construction, including wall porosity, thickness, texture and the presence or the absence of spines. Many of these fine morphological features have hypothesized (and in some cases, well-tested) functions, such as the use of pores for metabolic gas exchange, wall ornamentation for cytoplasmic anchoring and spines (or other surface ornamentation) for exposing symbionts to sunlight (in photosymbiotic taxa) and prey capture [60]. In future work, we aim to consider the importance of this omission through the inclusion of categorical species-specific fine morphological associations, but at present we have focused on what we can measure directly: gross morphology.

Gross morphology can vary dramatically among planktonic foraminifera, with species ranging from completely spherical (see *Orbulina universa* in figure 11), to pyramidal (see *T. truncatulinoides* in figure 5), to globular (see *N. dutertrei* in figure 5), discoidal (i.e. *Menardella menardii*), or digitate (i.e. finger-like projections in taxa like *Hastigerinella digitata*). In contrast to fine morphological features, surprisingly little is known about the functional importance of these gross morphological features. Earlier studies had often hypothesized hydrographic functions for these morphologies (as summarized in [34]), but these were recently elegantly laid to rest [33]. Given the importance of interspecific interactions in plankton communities [59], we suspect that interspecific associations (mutualistic or commensal), predation and oxygen limitation are likely the dominant forces shaping the evolution (and re-evolution) of gross morphology in planktonic foraminiferal lineages.

In this context, the analysis of the gross morphological similarities among 19 taxa (out of the roughly 40 morphological species globally) of modern planktonic foraminifera is particularly interesting for highlighting morphological affinities among taxa (figures 6 and 7*a*) and for exploring morphological, ecological and phylogenetic relationships (figure 12). Along the first two morphological axes (PC1 and PC2), the globorotaliform species cluster distinctly in the lower left quadrant, from the very flat *Menardella menardii* (most negative values along both axes) through the relatively inflated hirsutellids and *Globorotalia tumida*, to the umbilically domed truncorotaliids (figure 12). The relatively spherical taxa in this analysis (*Globigerinoides conglobatus*, *N. dutertrei*, *C. nitida*, *Globoconella inflata* and *P. obliquiloculata*) all cluster in the middle of PC1/PC2 space, with the relatively lobulate taxa along the edges with more positive PC1 (*Globigerina bulloides*, *Globigerinita glutinata*) or PC2 (*Globigerinoides sacculifur*, *Globigerinoides ruber*, *Globigerinella siphonifera*). Aside for the clustering among the taxonomically related globorotaliform taxa (along PC1 and PC2; figure 6), the phylogenetically mixed nature of morphospace occupation along the first two PCs is reinforced by the consensus clustering (figure 7*a*). Consensus clustering used all non-collinear PCs, which together account for more than 95% of the morphological variance. In the morphological consensus dendrogram, aside from two close taxonomic pairs (i.e. *Hirsutella* and *Neogloboquadrina*), the most striking feature of the dendrogram is the lack of apparent taxonomic or ecological cluster of the data, or obvious morphological affinities of the dominant clades—an observation supported by the distant relationship of full-3D morphology to both ecology or phylogeny alone (table 2). There appears to be more similarity between the ecological consensus dendrogram and the phylogeny than the morphological dendrogram and the phylogeny.

Rather than decoupling morphology from these two important influences (i.e. phylogeny and ecology), we suspect that this result emphasizes four aspects of planktonic foraminiferal evolution and morphology. First, major ecological features like depth habitat, biogeography and the presence/absence of symbionts appear to be relatively conservative. The relative similarity of the ecological and phylogenetic trees, and the tests of Pagel's *λ* on the three ecological characters (table 1) included in this study suggest that there is very high phylogenetic signal in these ecological traits, particularly for depth habitat and symbiont type. Second, in spite of widespread convergence in gross morphology [30,124], it is still unknown whether this convergence is functional or due to morphological constraints. It is also possible the most relevant ecological factors controlling morphology (like inter- and intraspecific interactions and metabolic demands) are the most poorly understood and therefore not considered. Recent studies (e.g. [125,126]) emphasize the importance of considering intraspecific variation in characterizing community ecology in the light of phenomena such as trait distribution overlap and niche packing. Third, our analysis is just a first attempt to explore this problem in planktonic foraminifera. For instance, we considered the relationships between morphology, ecology and phylogeny in just the 19 taxa with freely available 3D data via the Tohoku University Museum. Including all extant taxa (roughly 40–50 morphospecies) would provide a comprehensive test of the problem, as would including other time periods to explore the issue of morphological and ecological convergence. Fourth and finally, our method is designed to extract gross morphology and, as previously discussed, the traits used to segregate foraminifer species also include fine morphological features such as wall texture and spine presence/absence. These fine features are either impossible to extract by our method, or, if extractable, are not well resolved and therefore account for very little of the total morphological variation. By contrast, the gross morphological features that dominate the 3D shapes—including the most obvious first-order ones such as relative inflatedness/flatness—are often not highly informative taxonomically.

The tendency of gross morphology to capture many aspects of shape variation unrelated to ecology and phylogeny is unsurprising and would be a default expectation in many taxonomic groups. There are several reasons for this. First, even when functional morphology is well understood, there is no reason to expect a direct one-to-one mapping of morphology and ecology. In many cases, it is the interaction of multiple morphological features that underpins functionality, so a wide range of morphologies could have the same function. Second, gross morphology may poorly capture the underlying features that directly relate to ecological function or to phylogenetic relationships. For instance, ontogeny can greatly affect the first-order gross morphology, but the morphological differences between a juvenile and an adult (e.g. relative proportions, roundedness of features, number of segments and limbs, etc.) are not the features that are used to distinguish among species. For example, we suspect that a gross exterior morphological analysis of canids (e.g. dogs, wolves, foxes, coyotes, jackals, etc.) in varying ontological stages would likely find a large amount of variance related to the difference between adult and juvenile forms owing to the conspicuous differences between adult and juvenile builds and proportions (e.g. snout length, limb proportion, etc.). The importance of ontogeny to morphology is also well known in foraminifera, with juveniles showing a high degree of morphological conservatism when compared with adults [127–129]. Widespread morphological variation is well known intraspecifically as well in planktonic foraminifera—with, for instance, food availability dramatically influencing adult morphology in *T. sacculifer* [130]. In other words, the default expectation in a gross morphological analysis such as ours is that multiple factors are likely to influence shape, only some of which (and perhaps the least of which) are taxonomically or ecologically informative.

A practical implication of this phenomenon for principal component analyses of 3D morphology would be that the PCs capturing most of the variation may not be taxonomically informative. A first glance at the morphological phylogenies (figures 9, 10 and 12) would certainly seem to support this. To explore this idea further, we assessed the phylogenetic signal present in each individual PC in the semi- and full-3D datasets using Pagel's *λ*. We found four taxonomically informative PCs in the full 3D analysis (PCs 2, 4, 12 and 39) and six in the semi-3D analysis (PCs 2, 17, 96, 457, 555 and 588). In neither case was PC1 found to exhibit high phylogenetic signal, despite PC1 encompassing the highest amount of variation. However, PC2 was informative for both datasets, with a clear separation of the 3-chambered, globular *Globigerinoides ruber* and the bi-covex, multichambered *Globorotalia tumida* and *Menardella menardii* along the semi-3D PC2 (other morphological distinctions along phylogenetically informative PCs were difficult to interpret; see the electronic supplementary material, table S2). We repeated our clustering and tree distance analyses using only the most informative PCs for each dataset. For the full 3D-dataset, this resulted in greater congruence between the morphological and phylogenetic clusterings (PD = 32.019 versus 26.609). For the semi-3D dataset, there was little difference (PD = 24.429 versus 24.576).

### (c) Efficacy of semi-3D morphometrics: semi- and full-3D morphospaces

Semi- and full-3D approaches provide a very different perspective on gross morphological affinities between modern planktonic foraminifera (figure 10), with only the second being based upon the full-3D shape of individuals. For some questions, particularly those of size variation and morphospace occupation, the large differences in morphological space quantified by semi- and full-3D approaches may not matter. However, we generally think that methodological advances, similar to those described in §4*a* (towards high-throughput community dynamics), are needed to extract full-3D-like data from the semi-3D hulls. There are two reasons for this: first, some taxa have rather similar 3D shapes, like the pyramidal form of both *T. truncatulinoides* and *H. hirsuta*, which are reversed with regards to the umbilical/spiral axis. By ignoring half of an object's shape in semi-3D morphometrics, this inherent similarity is missed (compare figure 10*c* and *d* with regards to the inferred similarity of *T. truncatulinoides* and *H. hirsuta*). Second, the critical problem of viewing angle on inferred morphology in the semi-3D analyses is most easily addressed by projecting semi-3D morphologies into full 3D-shape space, rather than attempting to orient each half-hulled individual to the same perspective.

Given those caveats with regard to the current semi-3D morphological space, it is worth noting that the semi-3D data (after LDA) exhibits more structural similarity to the phylogenetic and ecological clustering than the full-3D shape space (table 2). This result is unexpected given the high resolution and completeness of the Tohoku University specimens compared to the semi-3D meshes extracted using our novel method. One possible reason for this result is the effective difference in sampling density between the full- and the semi-3D datasets. There are two aspects of sampling density that differ between the full- and semi-3D analyses. First, we examine hundreds as opposed to tens of individuals in the semi- versus full-3D analyses. By sampling more individuals in the semi-3D analysis, we likely captured more intra- and interspecific variation. Our results would thus suggest that it may be more important to optimize the number of individuals sampled over the quality of the 3D data, when such a trade-off must be made. Second, by using the same number of semi-landmarks (i.e. 256) for both the full- and the semi-3D meshes, we may have effectively under-sampled morphology in the full-3D dataset, as the 256 semi-landmarks are spread across the entire foraminifer rather than across only the top half of the foraminifer. In this sense, although the *raw* mesh resolution of the full-3D dataset is higher than that for the semi-3D dataset, the *effective* mesh resolution used for morphospace construction may actually be lower for the former than the latter. Further work is required in order to parse out these possible effects.

Regardless, it seems likely that for some questions, semi-3D shape spaces, like those briefly explored in figure 9*c*, might suffice for tracking community dynamics, although much future work is needed to explore this potential. By contrast, assemblage volume, discussed next, is a readily applicable trait, whose measurement is already enabled by the *run3dmorph* software.

### (d) Assemblage-wide ecometrics: quantification of community volume

Body size is a key determinant of interspecific interactions and alone can provide an important metric of biodiversity dynamics (and biotic drivers) through time [131]. For instance, assemblage dwarfing is one of the most coherent cross-clade responses to mass extinction and other abrupt perturbations (e.g. [132,133]). Similarly, over the course of the Cenozoic, there is a massive change in body size of well-fossilized marine plankton [131], with a dramatic increase in planktonic foraminiferal body size since the Miocene [108] and a Cenozoic-long decrease in lith size of coccolithophores [131,134]. Within extant assemblages, foraminiferal body size is correlated with latitude, with high latitudes corresponding to smaller average assemblage sizes and low latitudes corresponding to larger average assemblage sizes [135]. Even in the largest, most diverse modern assemblages in subtropical to tropical regions, adult body size separates otherwise co-occurring taxa [136], such that increasing assemblage diversity coincides with increasing size disparity.

Although many patterns and relationships have been observed, one limitation of most population-scale body size studies to-date is that the 2D silhouette is used as an implicit proxy for total size (i.e. volume or organic mass). In the case of foraminifera, and most shelly fauna, this is only approximately true. In planktonic foraminifera, for instance, relatively flattened morphologies are most common in the taxa with the largest 2D silhouettes. These taxa include most of the globorotaliform species and a number of other relatively un-inflated forms like *Globigerinella siphonifera* and *Trilobatus sacculifer*, all with higher surface area to volume ratios than are observed in relatively inflated taxa. Because of variation in the depth dimension, 3D measures of body volume are needed to check patterns inferred from 2D measures, a problem also discussed by Caromel *et al.* [127].

A first exploration of the relationship between 2D silhouette area and semi-3D volume in four North Atlantic assemblages (figure 13) reveals important differences between size distributions as inferred from 2D and 3D data. In both cases, the northern-most sites (CH 82-21 and EW 93-03-04) have the smallest maximum body sizes and a lower relative proportion of individuals in intermediate-large body sizes, with the northern-most site (EW 93-03-04) exhibiting smaller maximum body sizes than the next most northern site (CH 82-21). In the upper end of the size distributions, it is notable that the use of 3D body size estimates reduces the overall effect of this latitudinal trend on body mass. In both the 2D and the 3D data, the site with the largest body size (KC 78) is approximately fivefold larger than the site with the smallest (EW 93-03-04). If body volume increased equally in the depth dimension, however, the volumetric comparison (figure 13*b*) should have an 11-fold increase and not the roughly fivefold difference observed. In other words, tropical to subtropical assemblages are large, but they are very flat by comparison to high-latitude assemblages. This ‘flattening’ effect perhaps reflects the effects of metabolic constraints on morphology. While more data are certainly needed to explore the relationship between 2D silhouette size and body volume, this first analysis suggests that semi-3D volume data will be important for understanding the determinants of body size evolution through time, and for quantifying constraining inferences with regards to body mass distributions of populations in time and space. Excitingly, this semi-3D method can be seen as additive to the existing (much faster) 2D approaches, expanding rather than replacing the types of questions that can be addressed.

## 5. Conclusion

The main impetus for this study was to develop a high-throughput method for quantifying biotic dynamics in the abundant shelly-fossil record of life. To this end, we developed and presented the *run3dmorph* code for extracting 3D meshes and estimating surface area and volumes from light and microscopic images, tested the utility of planktonic foraminiferal shape as a proxy of community dynamics, and explored the importance of 3D measures of body size. Although this case study focused exclusively on planktonic foraminifera, *run3dmorph* is applicable for any object imaged with a series of *z*-stacks of known height. This broad applicability of *run3dmorph*, in combination with automated landmark-generation methods such as the Boyer *et al.* [91] *auto3dgm/PuenteAlignment* algorithm, is a step forward towards laying the groundwork for a ‘Next-Generation’ approach to morphometrics. Our semi-3D approach sacrifices data resolution and completeness relative to full-3D approaches like CT scanning, but has the advantages of accelerated processing speed, higher data density and reduced expense. Together, these improvements will allow a wider range of researchers to conduct robust analyses on population-level problems addressing the dynamics of 3D community size in time and space. At this point, much future work is still needed to make this semi-3D approach as robust and reliable as full-3D approaches to morphometrics, but the path to this ultimate goal is already clear.

For planktonic foraminifera, our early analyses of morphological patterns and body size variation are intriguing, as they suggest remarkably little similarity between overall gross morphology (in the Tohoku University specimens), phylogeny and certain aspects of ecology. This finding, combined with the pronounced ‘flattening’ of taxa towards tropical regions, emphasizes an exciting set of unknowns with regard to planktonic foraminiferal functional morphology, whose importance is underscored by the use of planktonic foraminifera as a palaeontological model species of macroevolution.

## Supplementary Material

Supplementary Figure 1 and overview

## Supplementary Material

Supplementary Table 1

## Supplementary Material

Supplementary Table 2

## Supplementary Material

Supplementary Table 3

## Supplementary Material

Supplementary Table 4

## Supplementary Material

Supplementary Table 5

## Supplementary Material

Supplementary Table 6

## Supplementary Material

Supplementary Table 7

## Supplementary Material

Supplementary Table 8

## Supplementary Material

Supplementary Table 9

## Data accessibility

The 3D mesh data (output from the Boyer *et al.* [91] automated landmark placement algorithm) are provided for the semi-3D images and Tohoku University Museum specimens in the electronic supplemental material, tables S3 and S4). Output from morphological analyses (e.g. principal component data and LDA data) is also provided in the electronic supplementary material, tables S5–S8. Low resolution Extended Depth of Focus (EDF) of the original slide scans are available through the Yale Peabody Museum (YPM) Division of Invertebrate Paleontology online catalogue (http://peabody.yale.edu/collections/search-collections?ip) by querying the Invertebrate Paleontology catalogue with the slide catalogue numbers (see list in the electronic supplementary material, table S9) and selecting the attached photo. The *AutoMorph* software package (including *segment, focus, run2dmorph* and *run3dmorph*) is available on GitHub at https://github.com/HullLab.

## References

[1] Losos JB, Baum DA, Futuyma DJ, Hoekstra HE, Lenski RE, Moore AJ, Peichel CL, Schluter D, Whitlock MC 2014 The Princeton guide to evolution, 880 p. Princeton, NJ: Princeton University Press.

[2] Foote M 1996 Perspective: evolutionary patterns in the fossil record. Evolution 50, 1–11. (10.2307/2410775) 28568887

[3] Venditti C, Meade A, Pagel M 2010 Phylogenies reveal new interpretation of speciation and the Red Queen. Nature 463, 349–352. (10.1038/nature08630) 20010607

[4] Jetz W, Thomas GH, Joy JB, Hartmann K, Mooers AO 2012 The global diversity of birds in space and time. Nature 491, 444–448. (10.1038/nature11631) 23123857

[5] Ezard THG, Aze T, Pearson PN, Purvis A 2011 Interplay between changing climate and species’ ecology drives macroevolutionary dynamics. Science 332, 349–351. (10.1126/Science.1203060) 21493859

[6] Purvis A, David C, Orme CDL, Toomey NH, Pearson PN 2009 Temporal patterns in diversification rates. In Speciation and patterns of diversity (eds Butlin RK, Bridle JR, Schluter D), pp. 278–300. Cambridge, UK: Cambridge University Press.

[7] Morlon H 2014 Phylogenetic approaches for studying diversification. Ecol. Lett. 17, 508–525. (10.1111/ele.12251) 24533923

[8] Peters SE, Kelly DC, Fraass AJ 2013 Oceanographic controls on the diversity and extinction of planktonic foraminifera. Nature 493, 398–401. (10.1038/nature11815) 23302802

[9] Lazarus D, Barron J, Renaudie J, Diver P, Turke A 2014 Cenozoic planktonic marine diatom diversity and correlation to climate change. PLoS ONE 9, pARTN e84857. (10.1371/journal.pone.0084857) PMC3898954 24465441

[10] Peters SE, Foote M 2002 Determinants of extinction in the fossil record. Nature 416, 420–424. (10.1038/416420a) 11919629

[11] Mayhew PJ, Bell MA, Benton TG, McGowan AJ 2012 Biodiversity tracks temperature over time. *Proc. Natl Acad. Sci. USA* 109, 15 141–15 145. (10.1073/pnas.1200844109) PMC3458383 22949697

[12] Quental TB, Marshall CR 2010 Diversity dynamics: molecular phylogenies need the fossil record. Trends Ecol. Evol. 25, 434–441. (10.1016/j.tree.2010.05.002) 20646780

[13] Lazarus DB 2011 The deep-sea microfossil record of macroevolutionary change in plankton and its study. In Comparing the geological and fossil records: implications for biodiversity studies, Geological Society Special Publication 358 (eds AJ McGowan and AB Smith), pp. 141–166. London, UK: The Geological Society of London.

[14] Foote M, Crampton JS, Beu AG, Marshall BA, Cooper RA, Maxwell PA, Matcham I 2007 Rise and fall of species occupancy in Cenozoic fossil mollusks. Science 318, 1131–1134. (10.1126/science.1146303) 18006744

[15] Foote M 2007 Symmetric waxing and waning of marine invertebrate genera. Paleobiology 33, 517–529. (10.1666/06084.1)

[16] Sexton PF, Norris RD 2008 Dispersal and biogeography of marine plankton: long-distance dispersal of the foraminifer *Truncorotalia truncatulinoides*. Geology 36, 899–902. (10.1130/G25232A.1)

[17] Kennett JP, Keller G, Srinivasan MS 1985 Miocene planktonic foraminiferal biogeography and paleoceanographic development of the Indo-Pacific region. In *The Miocene Ocean: paleoceanography and biogeography*, Geological Society of America Memoir 163 (ed. Kennett JP), pp. 197–236. Boulder, CO: The Geological Society of America.

[18] Pearson PN, Ezard THG 2014 Evolution and speciation in the Eocene planktonic foraminifer *Turborotalia*. Paleobiology 40, 130–143. (10.1666/13004)

[19] Hull PM, Norris RD 2009 Evidence for abrupt speciation in a classic case of gradual evolution. *Proc. Natl Acad. Sci. USA* 106, 21 224–21 229. (10.1073/pnas.0902887106) PMC2795541 19996180

[20] Vincent E, Berger WH 1981 Planktonic foraminifera and their use in paleoceanography. In The oceanic lithosphere (ed. Emiliani C), pp. 1025–1119. New York, NY: John Wiley & Sons, Inc.

[21] Kucera M 2007 Planktonic foraminifera as tracers of past oceanic environments. In Proxies in late Cenozoic paleoceanography (eds Hillaire-Marcel C, de Vernal A), pp. 213–262. Oxford, UK: Elsevier.

[22] Aze T, Ezard THG, Purvis A, Coxall HK, Stewart DRM, Wade BS, Pearson PN 2011 A phylogeny of Cenozoic macroperforate planktonic foraminifera from fossil data. Biol. Rev. 86, 900–927. (10.1111/j.1469-185X.2011.00178.x) 21492379

[23] Etienne RS, Haegeman B, Stadler T, Aze T, Pearson PN, Purvis A, Phillimore AB 2012 Diversity-dependence brings molecular phylogenies closer to agreement with the fossil record. Proc. R. Soc. B 279, 1300–1309. (10.1098/rspb.2011.1439) PMC3282358 21993508

[24] Wei KY, Kennett JP 1988 Phyletic gradualism and punctuated equilibrium in the late Neogene planktonic foraminiferal clade *Globoconella*. Paleobiology 14, 345–363. (10.1017/S0094837300012094)

[25] Fenton IS, Pearson PN, Dunkley Jones T, Farnsworth A, Lunt DJ, Markwick P, Purvis A 2016 The impact of Cenozoic cooling on assemblage diversity in planktonic foraminifera. Phil. Trans. R. Soc. B 371, 20150224 (10.1098/rstb.2015.0224) 26977064 PMC4810817

[26] Pearson PN 2012 Oxygen isotopes in foraminifera: overview and historical review. In Reconstructing earth's deep-time climate, Paleontological Society Papers series no. 12 (eds Ivany LC, Huber BT), pp. 1–38. Boulder, CO: The Paleontological Society.

[27] Moore TC Jr, Wade BS, Westerhold T, Erhardt AM, Coxall HK, Baldauf J, Wagner M 2014 Equatorial Pacific productivity changes near the Eocene-Oligocene boundary. Paleoceanography 29, 825–844. (10.1002/2014PA002656)

[28] Bolton CT, Gibbs SW, Wilson PA 2010 Evolution of nutricline dynamics in the equitorial Pacific during the late Pliocene. Paleoceanography 25, PA1207. (10.1029/2009PA001821)

[29] Holbourn A, Kuhnt W, Kochhann KGD, Andersen N, Sebastian Meier KJ 2015 Global perturbation of the carbon cycle at the onset of the Miocene Climatic Optimum. Geology 43, 123–126. (10.1130/G36317.1)

[30] Cifelli R 1969 Radiation of Cenozoic planktonic foraminifera. Syst. Zool. 18, 154–168. (10.2307/2412601)

[31] Coxall HK, Wilson PA, Pearson PN, Sexton PE 2007 Iterative evolution of digitate planktonic foraminifera. Paleobiology 33, 495–516. (10.1666/06034.1)

[32] Norris DR 1991 Parallel evolution in the keel structure of planktonic foraminifera. J. Foraminiferal Res. 21, 319–331. (10.2113/gsjfr.21.4.319)

[33] Caromel AGM, Schmidt DN, Phillips JC, Rayfield EJ 2014 Hydrodynamic constraints on the evolution and ecology of planktic foraminifera. Mar. Micropaleontol. 106, 69–78. (10.1016/J.Marmicro.2014.01.002)

[34] Lipps JH 1979 Ecology and paleoecology of planktic foraminifera. In Foraminiferal ecology and paleoecology, SEPM Short Course Notes *#*6 (eds Lipps JH, Berger WH, Buzas MA, Douglas RG, Ross CA), pp. 62–104. Houston, TX: Society of Economic Paleontologists & Mineralogists.

[35] Polly PD et al. 2011 History matters: ecometrics and integrative climate change biology. Proc. R. Soc. B 278, 1131–1140. (10.1098/rspb.2010.2233) PMC3049084 21227966

[36] Roy K, Foote M 1997 Morphological approaches to measuring biodiversity. Trends Ecol. Evol. 12, 277–281. (10.1016/S0169-5347(97)81026-9) 21238075

[37] Wainwright PC 2007 Functional versus morphological diversity in macroevolution. Annu. Rev. Ecol. Evol. Syst. 38, 381–401. (10.1146/annurev.ecolsys.38.091206.095706)

[38] Dietl GP 2013 The great opportunity to view stasis with an ecological lens. Palaeontology 56, 1239–1245. (10.1111/Pala.12059)

[39] Knappertsbusch M 2007 Morphological variability of *Globorotalia menardii* (planktonic foraminifera) in two DSDP cores from the Caribbean Sea and the Eastern Equatorial Pacific. Carnets Géol. 4, 1–34. (10.4267/2042/8455)

[40] Huber BT, Bijma J, Darling K 1997 Cryptic speciation in the living planktonic foraminifer *Globigerinella siphonifera* (d'Orbigny). Paleobiology 23, 33–62.

[41] Norris RD, Corfield RM, Cartlidge J 1996 What is gradualism? Cryptic speciation in globorotaliid foraminifera. Paleobiology 22, 386–405.

[42] Coxall HK 2000 Hantkeninid planktonic foraminifera and Eocene palaeoceanographic change, PhD dissertation, University of Bristol.

[43] Pearson PN, Coxall HK 2013 Origin of Eocene planktonic foraminifer Hantkenina by gradual evolution. Palaeontology 57, 243–267. (10.1111/pala.12064)

[44] Bé AWH, Spero HJ, Anderson OR 1982 Effects of symbiont elimination and reinfection on the life processes of the planktonic foraminifer *Globigerinoides sacculifer*. Mar. Biol. 70, 73–86. (10.1007/BF00397298)

[45] Kuroyanagi A, da Rocha RE, Bijma J, Spero HJ, Russell AD, Eggins SM, Kawahata H 2013 Effect of dissolved oxygen concentration on planktonic foraminifera through laboratory culture experiments and implications for oceanic anoxic events. Mar. Micropaleontol. 101, 28–32. (10.1016/J.Marmicro.2013.04.005)

[46] Spero HJ, Williams DF 1988 Extracting environmental information from planktonic foraminiferal δ^13^C data. Nature 335, 717–719. (10.1038/335717a0)

[47] Caron DA, Faber WW, Be AWH 1987 Effects of temperature and salinity on the growth and survival of the planktonic foraminifer *Globigerinoides sacculifer*. J. Mar. Biol. Assoc. UK 67, 323–341. (10.1017/S0025315400026643)

[48] Tohoku University Museum. 2008 e-Foram Stock. See http://webdb2.museum.tohoku.ac.jp/e-foram/.

[49] Kennett JP, Srinivasan MS 1983 Neogene planktonic foraminifera: a phylogenetic atlas. Stroudsburg, PA: Hutchinson Ross.

[50] Stewart DRM 2003 Evolution of Neogene globorotaliid foraminfiera and Miocene climate change. PhD dissertation, University of Bristol, UK.

[51] de Vargas C, Renaud S, Hilbrecht H, Pawlowski J 2001 Pleistocene adaptive radiation in *Globorotalia truncatulinoides*: genetic, morphologic, and environmental evidence. Paleobiology 27, 104–125. (10.1666/0094-8373(2001)027<0104:PARIGT>2.0.CO;2)

[52] Quillevere F, Morard R, Escarguel G, Douady CJ, Ujiie Y, de Garidel-Thoron T, de Vargas C 2013 Global scale same-specimen morpho-genetic analysis of *Truncorotalia truncatulinoides*: a perspective on the morphological species concept in planktonic foraminifera. Palaeogeogr. Palaeoclimatol. Palaeoecol. 391, 2–12. (10.1016/J.Palaeo.2011.03.013)

[53] Scott GH, Ingle JC Jr, McCane B, Powell CL II, Thunell RC 2015 *Truncorotalia crassaformis* from its type locality: comparison with Caribbean plankton and Pliocene relatives. Mar. Micropaleontol. 117, 1–12. (10.1016/j.marmico.2015.02.001)

[54] André A et al. 2013 The cryptic and the apparent reversed: lack of genetic differentiation within the morphologically diverse plexus of the planktonic foraminifer *Globigerinoides sacculifer*. Paleobiology 39, 21–39. (10.5061/Dryad.Rb06j)

[55] Spezzaferri S, Kucera M, Pearson PN, Wade BS, Rappo S, Poole CR, Morard R, Stalder C 2015 Fossil and genetic evidence for the polyphyletic nature of the planktonic foraminifera ‘*Globigerinoides*’, and description of the new genus *Trilobatus*. PLoS ONE 10, e0128108 (10.1371/journal.pone.0128108) 26020968 PMC4447400

[56] Darling KF, Kucera M, Kroon D, Wade CM 2006 A resolution for the coiling direction paradox in *Neogloboquadrina pachyderma*. Paleoceanography 21, PA2011. (10.1029/2005PA001189)

[57] Andre A, Quillevere F, Morard R, Ujiie Y, Escarguel G, de Vargas C, de Garidel-Thoron T, Douady CJ 2014 SSU rDNA Divergence in planktonic foraminifera: molecular taxonomy and biogeographic implications. PLoS ONE 9, ARTN e104641. (10.1371/journal.pone.0104641) PMC4131912 25119900

[58] Darling KF, Wade CA 2008 The genetic diversity of planktic foraminifera and the global distribution of ribosomal RNA genotypes. Mar. Micropaleontol. 67, 216–238. (10.1016/j.marmicro.2008.01.009)

[59] De Vargas C et al. 2015 Eukaryotic plankton diversity in the sunlit ocean. Science 348, 1261605 (10.1126/science.1261605) 25999516

[60] Hemleben C, Spindler M, Anderson OR 1989 Modern planktonic foraminifera. New York, NY: Springer.

[61] Oritz JD, Mix AC, Collier RW 1995 Environmental control of living symbiotic and asymbiotic foraminifera of the California Current. Paleoceanography 10, 987–1009. (10.1029/95PA02088)

[62] Fairbanks RG, Wiebe PH 1980 Foraminifera and chlorophyll maximum: vertical distribution, seasonal succession, and paleoceanographic significance. Science 209, 1524–1526. (10.1126/science.209.4464.1524) 17745963

[63] Fairbanks RG, Sverdlove M, Free R, Wiebe PH, Bé AWH 1982 Vertical distribution and isotopic fractionations of living planktonic foraminifera from the Panama Basin. Nature 298, 841–844. (10.1038/298841a0)

[64] Bé AWH, Bishop JKB, Svedlove MS, Gardner WD 1985 Standing stock, vertical distribution, and flux of planktonic foraminifera in the Panama Basin. Mar. Micropaleontol. 9, 307–333. (10.1016/0377-8398(85)90002-7)

[65] Friedrich O, Schiebel R, Wilson PA, Weldeab S, Beer CJ, Cooper MJ, Fiedig J 2012 Influence of test size, water depth, and ecology on Mg/Ca, Sr/Ca, *δ*18O and *δ*13C in nine modern species of planktic foraminifers. Earth Planet. Sci. Lett. 319, 133–145. (10.1016/j.epsl.2011.12.002)

[66] Pujol C, Vergnaud Grazzini C 1995 Distribution patterns of live planktic foraminifers as related to regional hydrography and productive systems of the Mediterranean Sea. Mar. Micropaleontol. 25, 187–217. (10.1016/0377-8398(95)00002-I)

[67] Gast RJ, Caron DA 2001 Photosymbiotic associations in planktonic foraminifera and radiolaria. Hydrobiologia 461, 1–7. (10.1023/A:1012710909023)

[68] Bé AWH, Tolderlund DS 1971 Distribution and ecology of living planktonic foraminifera in surface waters of the Atlantic and Indian Oceans. In The micropaleontology of marine bottom sediments (eds Funnell BM, Riedel WK), pp. 105–149. Cambridge, UK: Cambridge University Press.

[69] Berger WH 1969 Ecologic patterns of living planktonic foraminifera. Deep Sea Res. 16, 1–24. (10.1016/0011-7471(69)90047-3)

[70] Field DB 2004 Variability in vertical distributions of planktonic foraminifera in the California Current: relationships to vertical ocean structure. Paleoceanography 19, PA2014 (10.1029/2003PA000970)

[71] Ravelo AC, Fairbanks RG 1992 Oxygen isotopic composition of multiple species of planktonic foraminifera: reorders of the modern photic zone temperature gradient. Paleoceanography 6, 815–831. (10.1029/92PA02092)

[72] Bé AWH, Hutson WH 1977 Ecology of planktonic foraminifera and biogeographic patterns of life and fossil assemblages in the Indian Ocean. Micropaleontology 23, 369–414. (10.2307/1485406)

[73] Be AWH 1977 An ecological, zoogeographic, and taxonomic review of recent planktonic foraminifera. In Ocean micropalaeontology, (ed. Ramsay ATS), pp. 1–100 London, UK: Academic Press.

[74] Gastrich MD 1987 Ultrastructure of a new intracellular symbiotic algae found within planktonic foraminifera. J. Phycol. 23, 623–632. (10.1111/j.1529-8817.1987.tb04215.x)

[75] Schiebel R, Zeltner A, Treppke UF, Waniek JJ, Bollmann J, Rixen T, Hemleben C 2004 Distribution of diatoms, coccolithophores and planktic foraminifers along a trophic gradient during SW monsoon in the Arabian Sea. Mar. Micropaleontol. 51, 345–371. (10.1016/j.marmicro.2004.02.001)

[76] Birch H, Coxall HK, Pearson PN, Kroon D, O'Regan M 2013 Planktonic foraminifera stable isotopes and water column structure: disentangling ecological signals. Mar. Micropaleontol. 101, 127–145. (10.1016/j.marmicro.2013.02.002)

[77] Deuser WG, Ross EH 1989 Seasonally abundant planktonic formanifera of the Sargasso Sea: succession, deep-water fluxes, isotopic compositions, and paleoceanographic implications. J. Foraminifera Res. 19, 268–293. (10.2113/gsjfr.19.4.268)

[78] Cleroux C, de Monocal P, Arbuszewski J, Linsley B 2013 Reconstructing the upper water column thermal structure in the Atlantic Ocean. Paleoceanography 28, 1–14. (10.1002/palo.20050)

[79] Schiebel R, Hemleben C 2005 Modern planktic foraminifera. Palaontol. Z. 79, 135–148. (10.1007/BF03021758)

[80] Kasemann SA, Schmidt DN, Pearson PN, Hawkesworth CJ 2008 Biological and ecological insights into a Ca isotopes in planktic foraminifers as a paleotemperature proxy. Earth Planet. Sci. Lett. 271, 292–302. (10.1016/j.epsl.2008.04.007)

[81] Zerene Systems LLC (2015). http://zerenesystems.com/cms/stacker.

[82] Umorin M 2006 Stack Focuser. See http://rsb.info.nih.gov/ij/plugins/stack-focuser.html.

[83] Abramoff MD, Magalhaes PJ, Ram SJ 2004 Image processing with ImageJ. Biophoton. Int. 11, 36–42.

[84] Schneider CA, Rasband WS, Eliceiri KW 2012 NIH Image to ImageJ: 25 years of image analysis. Nat. Methods 9, 671–675. (10.1038/nmeth.2089) 22930834 PMC5554542

[85] Schindelin J et al. 2012 Fiji: an open-source platform for biological-image analysis. Nat. Methods 9, 676–682. (10.1038/nmeth.2019) 22743772 PMC3855844

[86] MATLAB. 2014 MATLAB and Statistics Toolbox Release 2014a. Natick, MA: The MathWorks, Inc.

[87] Körner S 2010 Export figure to 3D interactive PDF, MATLAB Central File Exchange. Retrieved 16 June 2015. See http://www.mathworks.com/matlabcentral/fileexchange/37640-export-figure-to-3d-interactive-pdf.

[88] Schwarz DM 2009 Matlab mesh to PDF with 3D interactive object, MATLAB Central File Exchange. Retrieved 16 June 2015. See http://www.mathworks.com/matlabcentral/fileexchange/25383-matlab-mesh-to-pdf-with-3d-interactive-object.

[89] Gramfort A 2010 Matlab mesh to PDF with 3D interactive object MATLAB Central File Exchange. Retrieved 16 June 2015. See http://www.mathworks.com/matlabcentral/fileexchange/25383-matlab-mesh-to-pdf-with-3d-interactive-object.

[90] Grahn A 2015 media9, CTAN Comprehensive TeX Archive Network. Retrieved 4 September 2015. See http://www.ctan.org/pkg/media9?lang=en.

[91] Boyer DM, Puente J, Gladman JT, Glynn C, Muhkerjee S, Yapuncich GS, Daubechies I 2015 A new fully automated approach for aligning and comparing shapes. Anatomic. Rec. 298, 249–276. (10.1002/ar.23084) 25529243

[92] R Core Team. 2013 R: a language and environment for statistical computing. Vienna, Austria: R Foundation for Statical Computing (http://www.R-project.org/)

[93] Glynn C, Puente J 2013 auto3dgm: 3-Dimensional geometric morphometrics. R software package version 1.0.

[94] Puente J 2013 Distances and algorithms to compare sets of shapes for automated biological morphometrics. PhD dissertation, Princeton University, NJ.

[95] Adams DC, Otarola-Castillo E 2013 geomorph: an R package for the collection and analysis of geometric morphometric shape data. Methods Ecol. Evol. 4, 393–399. (10.1111/2041-210X.12035)

[96] Gonzalez PN, Barbeito-Andrés J, D'Addona LA, Bernal V, Perez SI 2016 Technical note: Performance of semi and fully automated approaches for registration of 3D surface coordinates in geometric morphometric studies. Am. J. Phys. Anthropol. (10.1003/ajpa.22934) 26748891

[97] Paradis E, Claude J, Strimmer K 2004 APE: analyses of phylogenetics and evolution in R language. Bioinformatics 20, 289–290. (10.1093/bioinformatics/btg412) 14734327

[98] Ezard THG, Edgar KM, Hull PM 2015 Environmental and biological controls on size-specific δ^13^C and δ^18^O in recent planktonic foraminifera. Paleoceanography 30, 151–173. (10.1002/2014PA002735)

[99] Oksanen J (2015). http://CRAN.R-project.org/package=vegan.

[100] Venables WN, Ripley BD 2002 Modern applied statistics with S, 4th edn New York, NY: Springer.

[101] Steel MA, Penny D 1993 Distributions of tree comparison metrics: some new results. Syst. Biol. 42, 126–141.

[102] Schliep KP 2011 phangorn: phylogenetic analysis in R. Bioinformatics 27, 592–593. (10.1093/bioinformatics/btq706) 21169378 PMC3035803

[103] Wiens JJ, Donoghue MJ 2004 Historical biogeography, ecology and species richness. Trends Ecol. Evol. 19, 639–644. (10.1016/j.tree.2004.09.011) 16701326

[104] Ezard THG, Purvis A 2009 paleoPhylo: free software to draw paleobiological phylogenies. Paleobiology 35, 460–464. (10.1666/0094-8373-35.3.460)

[105] Revell LJ 2012 phytools: an R package for phylogenetic comparative biology (and other things). Methods Ecol. Evol. 3, 217–223. (10.1111/j.2041-210X.2011.00169.x)

[106] Smith FA et al. 2010 The evolution of maximum body size of terrestrial mammals. Science 330, 1216–1219. (10.1126/science.1194830) 21109666

[107] Heim NA, Knope ML, Schaal EK, Wang SC, Payne JL 2015 Cope's rule in the evolution of marine animals. Science 347, 867–870. (10.1126/science.1260065) 25700517

[108] Schmidt DN, Thierstein HR, Bollmann J, Schiebel R 2004 Abiotic forcing of plankton evolution in the Cenozoic. Science 303, 207–210. (10.1126/science.1090592) 14716007

[109] Cunningham JA, Rahman IA, Lautenschlager S, Rayfield EJ, Donoghue PCJ 2014 A virtual world of paleontology. Trends Ecol. Evol. 29, 347–357. (10.1016/J.Tree.2014.04.004) 24821516

[110] Mander L, Li M, Mio W, Fowlkes CC, Punyasena SW 2013 Classification of grass pollen through the quantitative analysis of surface ornamentation and texture. Proc. R. Soc. B 280, 20131905 (10.1098/rspb.2013.1905) PMC3779338 24048158

[111] Punyasena SW, Tcheng DK, Wesseln C, Mueller PG 2012 Classifying black and white spruce pollen using layered machine learning. New Phytol. 196, 937–944. (10.1111/J.1469-8137.2012.04291.X) 22943455

[112] Weller AF, Harris AJ, Ware JA 2006 Artificial neural networks as potential classification tools for dinoflagellate cyst images: a case using the self-organizing map clustering algorithm. Rev. Palaeobot. Palynol. 141, 287–302. (10.1016/J.Revpalbo.2006.06.001)

[113] Beaufort L, Dollfus D 2004 Automatic recognition of coccoliths by dynamical neural networks. Mar. Micropaleontol. 51, 57–73. (10.1016/J.Marmicro.2003.09.003)

[114] Grelaud M, Schimmelmann A, Beaufort L 2009 Coccolithophore response to climate and surface hydrography in Santa Barbara Basin, California, AD 1917–2004. Biogeosciences 6, 2025–2039. (10.5194/bg-6-2025-2009)

[115] Knappertsbusch MW, Binggeli D, Herzig A, Schmutz L, Stapfer S, Schneider C, Eisenecker J, Widmer L 2009 Amor—a new system for automated imaging of microfossils for morphometric analyses. Palaeontol. Electron. 12.

[116] Görög A, Szinger B, Toth E, Viszkok J 2012 Methodology of the micro-computer tomography on foraminifera. Palaeontol. Electron. 15 See http://content/content/pdfs/261.pdf.

[117] Foster LC, Schmidt DN, Thomas E, Arndt S, Ridgwell A 2013 Surviving rapid climate change in the deep sea during the Paleogene hyperthermals. *Proc. Natl Acad. Sci. USA* 110, 9273–9276. (10.1073/pnas.1300579110) PMC3677492 23690593

[118] Hull PM, Norris RD, Bralower TJ, Schueth JD 2011 A role for chance in marine recovery from the end-Cretaceous extinction. Nat. Geosci. 4, 856–860. (10.1038/ngeo1302)

[119] Bollmann J et al. 2004 Automated particle analysis: calcarous microfossils. In Image analysis, sediments, and paleoenvironments (ed. Francus P), pp. 229–252. Dordrecht, The Netherlands: Kluwer Academic Publishers.

[120] Bralower T, Eccles L, Kutz J, Yancey T, Schueth J, Arthur M, Bice D 2010 Grain size of Cretaceous–Paleogene boundary sediments from Chicxulub to the open ocean: implications for interpretation of the mass extinction event. Geology 38, 199–202. (10.1130/G30513.1)

[121] Ezard THG, Quental TB, Benton MJ 2016 The challenges to inferring the regulators of biodiversity in deep time. Phil. Trans. R. Soc. B 371, 20150216 (10.1098/rstb.2015.0216) 26977058 PMC4810811

[122] Brown JH, Gillooly JF, Allen AP, Savage VM, West GB 2004 Toward a metabolic theory of ecology. Ecology 85, 1771–1789. (10.1890/03-9000)

[123] Schmidt-Nielsen K 1984 Scaling: why is animal size so important? Cambridge, UK: Cambridge University Press.

[124] Norris RD 1991 Biased extinction and evolutionary trends. Paleobiology 17, 388–399.

[125] Bolnick DI et al. 2011 Why intraspecific trait variation matters in community ecology. Trends Ecol. Evol. 26, 183–192. (10.1016/j.tree.2011.01.009) 21367482 PMC3088364

[126] Violle C, Enquist BJ, McGill BJ, Jiang L, Albert CH, Hulshof C, Jung V, Messier J 2012 The return of variance: intraspecific variability in community ecology. Trends Ecol. Evol. 27, 244–252. (10.1016/j.tree.2011.11.014) 22244797

[127] Caromel AGM, Schmidt DN, Fletcher I, Rayfield EJ 2016 Morphological change during the ontogeny of the planktic foraminifera. J. Micropalaeontol. 35, 2–19. (10.1144/jmpaleo2014-017)

[128] Brummer G-JA, Hemleben C, Spindler M 1986 Planktonic foraminiferal ontogeny and new perspectives for micropalaeontology. Nature 319, 50–52. (10.1038/319050a0)

[129] Brummer G-JA, Hemleben C, Spindler M 1987 Ontogeny of extant spinose planktonic foraminifera (Globigerinidae): a concept exemplified by *Globigerinoides sacculifer* (Brady) and *G. ruber* (D'Orbigny). Mar. Micropaleontol. 12, 357–381. (10.1016/0377-8398(87)90028-4)

[130] Bé AWH, Caron DA, Anderson OR 1981 Effects of feeding frequency on life processes on the planktonic foraminifer *Globigerinoides sacculifer* in laboratory culture. J. Mar. Biol. Assoc. UK 61, 257–277. (10.1017/S002531540004604X)

[131] Schmidt DN, Lazarus D, Young JR, Kucera M 2006 Biogeography and evolution of body size in marine plankton. Earth-Sci. Rev. 78, 239–266. (10.1016/j.earscirev.2006.05.004)

[132] Twitchett RJ 2007 The Lilliput effect in the aftermath of the end-Permian extinction event. Palaeogeogr. Palaeoclimatol. Palaeoecol. 252, 132–144. (10.1016/J.Palaeo.2006.11.038)

[133] McInerney FA, Wing SL 2011 The Paleocene-Eocene Thermal Maximum: a perturbation of carbon cycle, climate, and biosphere with implications for the future. Annu. Rev. Earth Planet. Sci. 39, 489–516. (10.1146/Annurev-Earth-040610-133431)

[134] Herrmann S, Thierstein HR 2012 Cenozoic coccolith size changes—evolutionary and/or ecological controls? Palaeogeogr. Palaeoclimatol. Palaeoecol. 333, 92–106 (10.1016/j.palaeo.2012.03.011)

[135] Schmidt DN, Renaud S, Bollmann J, Schiebel R, Thierstein HR 2004 Size distribution of Holocene planktic foraminifer assemblages: biogeography, ecology and adaptation. Mar. Micropaleontol. 50, 319–338. (10.1016/S0377-8398(03)00098-7)

[136] Al-Sabouni N, Kucera M, Schmidt DN 2007 Vertical niche separation control of diversity and size disparity in planktonic foraminifera. Mar. Micropaleontol. 63, 75–90. (10.1016/j.marmicro.2006.11.002)

