# Supplementary material for: Towards a morphological metric of assemblage dynamics in the fossil record: a test case using planktonic foraminifera
Source: Philos Trans R Soc Lond B Biol Sci. 2016 Apr 5;371(1691):20150227. doi: 10.1098/rstb.2015.0227 (PMC4810820; doi:10.1098/rstb.2015.0227)
Supplement: Supplementary Figure 1 and overview [file rstb20150227supp1.pdf]

Supplementary material for Hsiang AY, Elder LE, Hull PM (2016) Towards a morphological metric of assemblage dynamics in the fossil record: A test case using planktonic foraminifera, *Phil. Trans. R. Soc. B.* doi: 10.1098/rstb.2015.0227

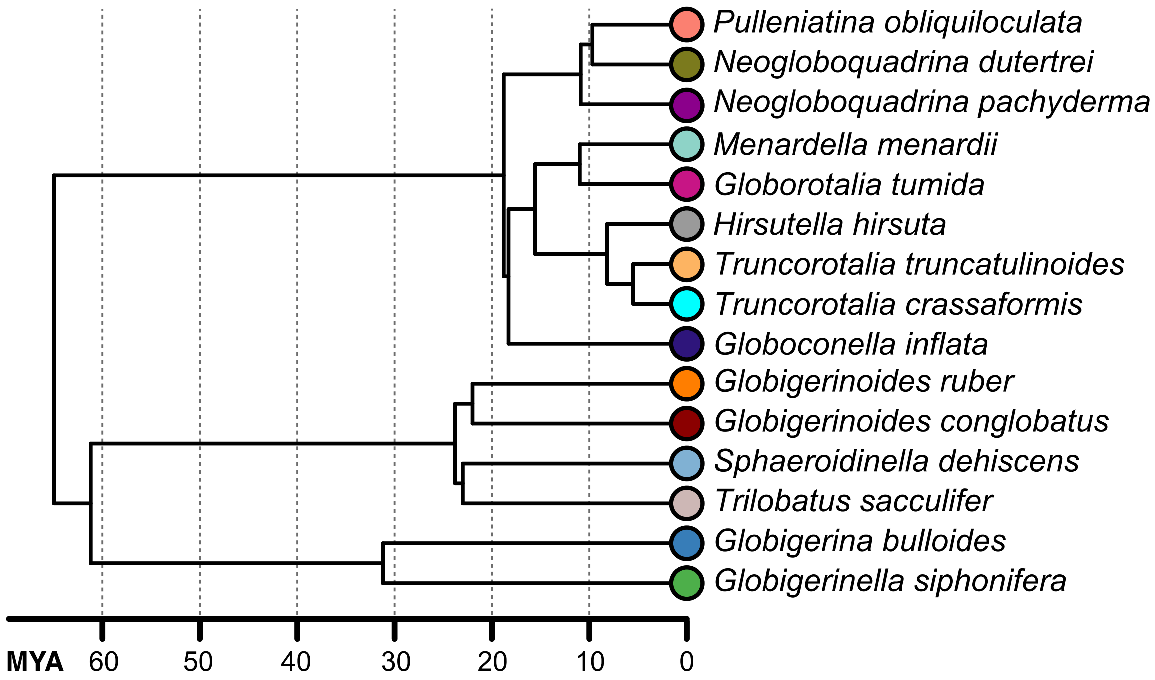

Supplementary Figure 1: Time-calibrated morphological phylogeny of macroperforate planktonic foraminifera from Aze *et al.* (2011), pruned to include only the fifteen species that overlap between the semi-3D and full-3D datasets (excluding the sixteenth overlapping species, the microperforate *Candeina nitida*.

<u>Supplementary tables supplied separately</u>

Supplementary Table 1: Complete list of Yale Peabody Museum (YPM) and Tohoku University Museum e-Foram Stock specimens used for semi-3D and full-3D morphospace analyses, including accession numbers and species identifications (when available).

Supplementary Table 2: Results of assessment of phylogenetic signal present in every individual principle component (PC) using Pagel's λ in the full-3D (a-c) and semi-3D (d-f) datasets. (a,c) PCs with high phylogenetic signal ($\lambda > 0.5$ and $p < 0.05$) for the Tohoku University dataset and semi-3D dataset, respectively; (b,d) List of five minimum and maximum individuals along the first three high PC axes from (a) (PC2, 4, and 12) and (c) (PC2, 17, and 96); (c,f) Path difference tree distance as calculated against consensus dendrograms built from: (c) PC4 only, all high PCs from (a) combined, and the Aze *et al.* phylogeny; (f) PC2 only, all high PCs from (c) combined, and the Aze *et al.* phylogeny.

Supplementary Table 3: Output from Boyer *et al.* (2015) automated landmark placement algorithm for the semi-3D dataset, in *Morphologika* format.

Supplementary Table 4: Output from Boyer *et al.* (2015) automated landmark placement algorithm for the Tohoku University Museum full-3D dataset, in *Morphologika* format.

Supplementary Table 5: Principle component analysis (PCA) results using the semi-3D dataset, including all 597 PCs.

Supplementary Table 6: Principle component analysis (PCA) results using the full-3D dataset, including all 39 PCs.

© Hsiang AY, Elder LE, and Hull PM under the terms of the Creative Commons Attribution License http://creativecommons.org/licenses/by/3.0/, which permits unrestricted use, provided the original author and source are credited.

Supplementary material for Hsiang AY, Elder LE, Hull PM (2016) Towards a morphological metric of assemblage dynamics in the fossil record: A test case using planktonic foraminifera, *Phil. Trans. R. Soc. B.* doi: 10.1098/rstb.2015.0227

Supplementary Table 7: Results of the linear discriminant analysis using the principle components (PCA-LDA) for the semi-3D dataset, including all 15 linear discriminant axes, for the species-identified individuals and reproducibility test 'individuals' (421 total).

Supplementary Table 8: Results of the linear discriminant analysis using the principle components (PCA-LDA) for the semi-3D dataset, including all 15 linear discriminant axes, for the additional 116 well preserved coretop individuals from KC78, EW93-03, AII 42/2/2, and CH82-21.

Supplementary Table 9: List of Yale Peabody Museum catalogue numbers for all slides included in this study. These numbers can be used to obtain low resolution 2D EDF images from the Yale Peabody Museum catalogue.

References

Aze, T., Ezard, T.H.G., Purvis, A., Coxall, H.K., Stewart, D.R.M., Wade, B.S. & Pearson, P.N. 2011 A phylogeny of Cenozoic macroperforate planktonic foraminifera from fossil data. *Biological Reviews* **86**, 900-927. (doi: 10.1111/j.1469-185X.2011.00178.x)

Boyer, D.M., Puente, J., Gladman, J.T., Glynn, C., Muhkerjee, S., Yapuncich, G.S. & Daubechies, I. 2015 A new fully automated approach for aligning and comparing shapes. *Anatomical Record* **298**, 249- 276. (doi: 10.1002/ar.23084)

© Hsiang AY, Elder LE, and Hull PM under the terms of the Creative Commons Attribution License http://creativecommons.org/licenses/by/3.0/, which permits unrestricted use, provided the original author and source are credited.
